# Physiological and Proteomic Changes in the Apoplast Accompany Leaf Senescence in *Arabidopsis*

**DOI:** 10.3389/fpls.2019.01635

**Published:** 2020-01-08

**Authors:** Maria L. Borniego, Maria C. Molina, Juan J. Guiamét, Dana E. Martinez

**Affiliations:** Instituto de Fisiología Vegetal (INFIVE), CONICET-Universidad Nacional de La Plata, La Plata, Argentina

**Keywords:** apoplast, senescence, apoplastic fluid, secretome, extracellular pH, pathogenesis-related protein, PR

## Abstract

The apoplast, i.e. the cellular compartment external to the plasma membrane, undergoes important changes during senescence. Apoplastic fluid volume increases quite significantly in senescing leaves, thereby diluting its contents. Its pH elevates by about 0.8 units, similar to the apoplast alkalization in response to abiotic stresses. The levels of 159 proteins decrease, whereas 24 proteins increase in relative abundance in the apoplast of senescing leaves. Around half of the apoplastic proteins of non-senescent leaves contain a N-terminal signal peptide for secretion, while all the identified senescence-associated apoplastic proteins contain the signal peptide. Several of the apoplastic proteins that accumulate during senescence also accumulate in stress responses, suggesting that the apoplast may constitute a compartment where developmental and stress-related programs overlap. Other senescence-related apoplastic proteins are involved in cell wall modifications, proteolysis, carbohydrate, ROS and amino acid metabolism, signaling, lipid transport, etc. The most abundant senescence-associated apoplastic proteins, PR2 and PR5 (e.g. pathogenesis related proteins PR2 and PR5) are related to leaf aging rather than to the chloroplast degradation program, as their levels increase only in leaves undergoing developmental senescence, but not in dark-induced senescent leaves. Changes in the apoplastic space may be relevant for signaling and molecular trafficking underlying senescence.

## Introduction

At the leaf level, senescence represents a degenerative process, but, at the same time it constitutes an efficient nutrient recycling mechanism for the plant. Most of the nitrogen released from senescing leaves comes from Rubisco and other plastid proteins degradation; therefore a major focus of senescence research has been to elucidate the mechanism for chloroplast dismantling (Noodén et al., 1997; Guiamet et al., 2002; Otegui, 2018). Other cellular changes occurring in senescing leaves are less characterized, and even less is known about the adjustments taking place outside the plasma membrane as leaf senescence progresses.

The apoplast is the space external to the plasma membrane that includes cell walls, middle lamella, intercellular spaces, and the fluid that moves freely within the walls and intercellular spaces, known as extracellular fluid or apoplastic fluid (AF). The AF represents around 4% to 11% of the total leaf fresh weight (Sattelmacher and Horst, 2007). Its composition consists of molecules related to metabolism and signaling, such as amino acids, polysaccharides, secondary metabolites, ions, and secreted mRNA and proteins with several functions, particularly enzymes related to defense and proteolysis (Sattelmacher and Horst, 2007; Martínez‐González et al., 2018). The apoplast plays a critical role in plant development, as it provides the environment for cell expansion and cell wall maintenance, ion and molecule trafficking, source-to-sink translocation of nutrients, intercellular and systemic signaling, stress perception, and response (Hoson, 1998; O'Leary et al., 2016; Rodríguez-Celma et al., 2016). Stress-elicited changes in the apoplast have been extensively examined, and these include modulation of the pH and fine tuning of protein abundance and composition (Felle, 2001; Dani et al., 2005; Floerl et al., 2012; Geilfus, 2017). Apoplastic pH (pH_apo_) transiently increased under drought and salinity becoming more alkaline as the stresses intensify (Geilfus et al., 2015; Zörb et al., 2015; Geilfus, 2017). Also, pathogen and peptide signals, such as systemin and RALF (rapid alkanilization factor), trigger apoplast alkalization (Felle et al., 2004; Higgins et al., 2007; Masachis et al., 2016). The pH_apo_ varies along ontogeny (Mattsson and Schjoerring, 2003; Wu et al., 2016), and along with pH_apo,_ the extracellular space volume might adjust as the leaf ages (Husted and Schjoerring, 1995; Lohaus et al., 2001; Nouchi et al., 2012). Apoplast conditions influence chloroplast metabolism and leaf physiology (Karpinska et al., 2018) and therefore might influence senescence-related processes, for instance the regulation of intra to extracellular exchange of molecules and long distance remobilization of nutrients. Transcription analysis of *Arabidopsis* plasma membrane transporters suggest that molecule trafficking across the plasma membrane might increase during leaf senescence (Van Der Graaff et al., 2006).

Proteomic approaches identified chitinases, pathogenesis related proteins (PR), and other defense related enzymes, as the mayor leaf apoplastic proteins (Boudart et al., 2005; Rutter and Innes, 2017; Soares et al., 2017). The extracellular accumulation of these enzymes along with the transient alkalinization of pH_apo_ as signature of different biotic and abiotic stress responses suggest either a cross-talk between stress pathways or a common apoplastic signal-transducing element or node (Geilfus, 2017).

Many of the stress-related extracellular enzymes are constitutive members in the AF that activate and/or accumulate upon specific signals, some of them are also up-regulated during senescence (Grudkowska and Zagdanska, 2004; Goulet et al., 2010). Other stress-related enzymes relocate inside/outside the cell in response to external stimulus. Caspase-like serine proteases from *Avena sativa* relocate from the cytosol to the apoplast upon programmed cell death (PCD) induction (Coffeen and Wolpert, 2004), whereas some apoplastic subtilisin proteases re-enter cells committed to PCD (Trusova et al., 2019).

Similar intra-extracellular pathways might be involved in the regulation and/or execution of leaf senescence.

Compared to stress-related broad analysis of AF proteomes (Kosová et al., 2011; Gupta et al., 2015), there is not enough information on the AF proteome dynamics in senescing leaves, however different studies evidence relevant roles for apoplastic proteins during this leaf stage. By regulating long-distance movement of sucrose, the extracellular invertase (cwINV) and its inhibitor (INVINH) probably play a crucial role in the regulation of senescence by controlling source-sink relations (Lara et al., 2004; Jin et al., 2009). The apoplastic subtilisin protease SASP is highly up-regulated during senescence, and whereas at-*sasp* plants do not differ from wild type plants at vegetative stage, they produce more branched inflorescences, siliques, and seeds (Martinez et al., 2015). The *Arabidopsis* extracellular metalloprotease At2-MMP is up-regulated as the plant ages, and at2-*mmp-1* plants show accelerated chlorophyll (Chl) degradation and delayed flowering (Delorme et al., 2000; Golldack et al., 2002). Other apoplastic proteases from different mechanistic classes (cysteine-, metallo-, and serine- proteases) are up-regulated during leaf senescence (Martínez and Guiamet, 2014).

This study aimed to shed light on the dynamics of the extracellular space during leaf senescence by analyzing physiological parameters of the apoplast space and the AF, including a large-scale quantitative proteomic approach to compare the AF proteomes of senescent and non-senescent leaves.

## Materials and Methods

### Plant Material and Growth Conditions

*Arabidopsis thaliana* Col-0, wild type, and the transgenic line apo-pHusion (Gjetting et al., 2012) were used. Apo-pHusion plants express the chimeric apo-mRFP1-EGFP protein targeted to the apoplast, where it functions as a pH sensor (Gjetting et al., 2012). The plants were cultivated in 550 mL pots filled with soil and vermiculite (2:1 v/v). Nitrofoska^®^ was applied (30 mL, 1 g/L per pot) every 30 days. The plants were grown in growth chambers, at 24°C and 120 μmol m^−2^ s^−1^ photosynthetic photon flux density under a 10 h light/14 h dark photoperiod.

Each rosette was separated in groups of leaves based on the phyllotaxis and leaf size. Vegetative rosettes were separated in two groups of leaves, the youngest named S1 (Stage 1), whereas rosettes at reproductive stages were separated in three, four, or five groups of leaves, according to the rosette age and leaf number (stages S2, S3, and S4). Around six to eight consecutive leaves representing one stage (S1, 2, 3, or 4) were sampled per plant. Each plant was harvested only once and for one particular Stage only.

### Physiological Parameters

*Leaf chlorophyll content* was measured non-destructively with the SPAD 502 Portable Chlorophyll Meter (Konica-Minolta^®^).

Maximum quantum yield of photosystem II (Fv/Fm) measurements were taken with a modulated pulse fluorimeter FMS 2 (Hansatech^®^). The leaves were acclimated for 30 min in the dark before the measurements.

*Leaf water content*, LWC, was determined as:

LWC = [(FW − DW)/FW]×100

*Relative water content,* RWC, was determined as:

RWC = [(FW−DW)/(SW−DW)]×100

where FW = fresh weight, DW = dry weight, SW = water saturated weight.

*Water potential (Ψ_w_)* of 5 mm diameter leaf discs was measured with a dew point psychrometer and C-52 chambers (Wescor^®^). Sodium chloride solutions of different concentrations were absorbed in filter paper discs and used for calibration.

*Solute potential (Ψs)*: measurements were carried out in a vapor pressure micro osmometer Vapro 5520 (Wescor^®^), using 10 μL per sample. To measure leaf Ψs, leaves without the midrib were frozen in liquid nitrogen and placed in a 1 mL syringe containing glass wool at the bottom. Pressure was applied pushing the plunger and the resulting leaf macerates were analyzed. The Ψs of the AF was estimated measuring the Ψs of the apoplastic wash fluids (AWFs), and correcting the obtained values by their corresponding dilution factors (F_dil_) (See below).

### Leaf AWF Extraction

Leaf infiltration was performed with cold deionized water using a 60 mL syringe as described in O'Leary et al. (2014). Infiltrated leaves were stuck, with petioles facing up into a 50-mL centrifuge tube and centrifuged at 600 g for 40 min at 4°C. For protein analysis, 5 μL of 300 mM phenylmethylsulfonyl fluoride (PMSF) was added at the bottom of the tube prior to centrifugation.

### Estimation of the Apoplast Volume Occupied by Fluid (V_AF_) and Air (V_air_) and of AWF Dilution Factor

Estimations of V_air_ and V_AF_ were carried out by the Indigo Carmine (indigo-5.5′disulfonic acid, disodium salt, IC) method (Husted and Schjoerring, 1995) with modifications. Fully hydrated leaves were excised from *Arabidopsis* plants, weighed, and vacuum infiltrated, as described above, but with 50 μM IC, in 10 mM sodium phosphate buffer pH 6.2. Once infiltrated, leaf surfaces were blotted, immediately re-weighed, and centrifuged. The difference in the leaf weight before and after infiltration (corrected by the density of IC solution) was used for calculation of the apoplastic air volume (V_air_) (Lohaus et al., 2001).

The IC infiltration solution and AWFs were measured spectrophotometrically at 610 nm to calculate the IC concentration. The volume of AF (V_AF_) was calculated using the following equation:

$$V_{AF}=\;\left(V_{air}\times {Abs}_{610}IC\right)/{Abs}_{610}AWF)-V_{air}$$

The *dilution factor (F_dil_)* of AWF was calculated based on V_AF_ and V_air_:

$${\text{F}}_{\text{dil}}({\text{V}}_{\text{AF}}+{\text{V}}_{\text{air}})/{\text{V}}_{\text{AF}}$$

The physiological concentration of solutes and metabolites in the AF was calculated by multiplying the solute or metabolite concentration in the AWF by its *dilution factor*, F_dil_.

### Cytoplasmic Contamination in Apoplast Fluids

The cytosolic marker glucose-6-phosphate dehydrogenase (G6PDH, EC 1.1.1.49) was assayed at room temperature in 100 µL 100 mM Tris-HCl pH 8, 6.7 mM MgCl_2_, 12 mM glucose-6-P, 0.4 mM NADP^+^ and AWF or leaf extracts. NADP^+^ reduction was followed at 340 nm. Leaf extracts were obtained by homogenizing leaves at 4°C in 100 mM Tris-HCl pH 8, 4 mM PMSF; and centrifuging for 15 min at 15,000 g, 4°C.

### *In Vivo* Estimation of Leaf Apoplast pH by Confocal Fluorescence Microscopy

#### Ex Vivo Calibration of pHusion

Aliquots of AWF extracted from apo-pHusion plants were incubated with 300 mM Tris-HCl or 300 mM MES buffers adjusted with HCl or KOH to different pH values, placed in microscope slides with cavities, and detected by confocal microscopy. Confocal data acquisition was performed with a Leica TCS SP5 II CLSM with the following setting: Ex 488/Em 524–550 nm for EGFP, and Ex 543/Em 566–634 nm for mRFP1. For each pH value, pixel intensity of the fluorescence ratio EGFP/mRFP1 in a region of interest (ROI) was calculated using the software ImageJ. A non-linear fitting of average pixel intensities was calculated using ImageJ software, and the software GraphPad Prism was used to generate a sigmoid curve.

#### *In Vivo* Observation of Apoplastic Apo-pHusion

Images of apo-pHusion S2 and S3 leaves were acquired by confocal microscopy, with the setting described above. Ratio images EGFP/mRFP1 were generated using the software ImageJ and the average pixel intensities EGFP/mRFP1 for each ROI were calculated. PH values were estimated by extrapolation on the calibration curve. LAS AF software version 2.2.1 was used for image acquisition. For visual presentation of ratio images, a pseudocolor look-up table was designed using the software ImageJ.

### Isolation of Cell Wall Polysaccharides and Cell Wall Quantitation

Cell wall polysaccharides were obtained from alcohol insoluble residues (AIR) according to Nardi et al. (2015). To estimate the amount of cell wall, the starch was subtracted from each AIR with Dimethyl sulfoxide (DMSO) according to Carpita and Kanabus (1987). After removing the starch, the resulting AIR was incubated with 0.01 M HCl in a boiling water bath for 2 h and filtered for polyuronide extraction. The obtained solution was used for pectin quantification (see below).

#### Hydrolysis and Quantification of Pectins

100 μL of the polyuronide filtrate was mixed with 600 μL of 0.0125 M sodium tetraborate in saturated H_2_SO_4_, incubated in a water bath for 20 min. and cooled down. Uronic acid concentrations were estimated by the m-hydroxydiphenyl method (Blumenkrantz and Asboe-Hansen, 1973) using galacturonic acid as standard.

#### Starch Quantification

Around 0.1 mL of DMSO extract was mixed with 1 mL of 0.05% w/v anthrone in 66% v/v H_2_SO_4_ and incubated at 100°C for 10 min and cooled down. The starch content was measured spectrophotometrically at 620 nm using glucose as standard.

### Transmission Electron Microscopy

Around 2 mm^2^ pieces were sectioned from the middle region of the leaf and fixed by immersion in 2% glutaraldehyde, post fixed in 1% osmium tetroxide and dehydrated through a graded series of ethanol and embedded in epoxy resin. Ultrathin sections were cut with a Super Nova Reichert–Jung Ultra-microtome (Wien Austria), and examined with a JEM 1200 EX II transmission electron microscope (JEOL Ltd., Tokio, Japan). Cell wall analysis was performed with Image J software on at least three different leaves of each stage.

### Protein Analysis

Leaves were homogenized in 50 mM Tris-HCl pH 8, 20 mM ethylenediaminetetraacetic acid (EDTA), 10 mM PMSF and 1% insoluble polyvinylpyrrolidone, and cleared at 15,000 g, 4°C for 15 min. Laemmli's Sample Buffer, LSB, was added before electrophoresis. Aliquots of AWF were lyophilized in a vacuum freeze-dryer (Scientz^®^-10 N) and the pellets were re-suspended in LSB.

SDS-PAGE gels (12% w/v acrylamide) were run in a Mini-PROTEAN^®^ III cell (Bio-Rad), and stained with Coomassie-Brilliant Blue R-250 (CBB) or with Coomassie Blue Colloidal G-250 (G-250).

Densitometric quantification of protein bands in CBB-stained gels was performed by using the ImageJ 1.6 software (National Institutes of Health, USA).

#### Peptide and Protein Quantification

The Pierce^®^ BCA Protein Assay Kit (Thermo Scientific) using bovine serum albumin as standard was used with AWFs and leaf extracts

#### Western Blot and Immunodetection

SDS-PAGE gels were transferred to nitrocellulose membranes using a Bio Rad Miniprotean System. The membrane was blocked with 10% milk in PBST buffer, incubated with a polyclonal antibody against Rubisco Large Subunit, and developed with a horseradish *peroxidase*-conjugated secondary antibody.

### Protein Identification and Quantification by LC-MS/MS and Bioinformatics

For the identification of proteins in SDS-PAGE gels the bands were excised from G-250 stained gels, washed, dried, reduced, alkylated, and trypsin digested. Peptides were extracted with three steps of 50% acetonitrile/0.5% trifluoroacetic acid, and desalted with a Zip-Tip C18. Samples were analyzed by LC-MSMS using a Q Exactive™ nano HPLC-ESI-Orbitrap mass spectrometer (Thermo Fisher Scientific). Protein identification was performed with Proteome Discoverer 1.4 (Thermo Fisher Scientific) connected to a Mascot search engine server (Matrix Science, London, UK). Only proteins identified with at least two unique peptides with 0.99 confidence (FDR = 0.01) were considered.

For large-scale protein analysis by shotgun proteomics, lyophilized AWF aliquots were resuspended in 8 M urea; aliquots containing 20 µg of protein were reduced, alkylated, trypsin digested, and desalted. LC-MSMS analysis was performed as described previously. A *label-free-quantification* method was performed using Proteome Discoverer 1.4. Four biological replicas for each leaf stage were analyzed. Identified proteins were submitted to a Student's t-test (*P* ≤ 0.05) with Perseus 1.6.1.3 (Max Planck Institute of Biochemistry). Only proteins exhibiting at least a ±100% fold change in their amount were considered.

### Statistical Analysis

Data from parameters (Chl, protein content) were tested either with one-way ANOVA and Tukey tests (*P* ≤ 0.05), with two-way ANOVA and Holm-Sidak test (p ≤ 0.05), or with Dunnett's multiple comparisons test. For paired analysis of S2 and S3 leaf stages, means were compared using the Student's t-test (p ≤ 0.05). Statistical analysis was plotted using the GraphPad Prism 6.01 software (GraphPad Software, San Diego, CA, USA). The number of independent replicates, i.e, biological samples (n), and the corresponding technical replicates are indicated in each table and figure caption.

Cell wall thickness was analyzed by one-way ANOVA, and univariate tests of significance, Sigma-restricted parameterization Effective hypothesis decomposition (*P* ≤ 0.0001).

## Results and Discussion

### Leaf Developmental Stages and Apoplastic Fluid Isolation

**Figure 1:**
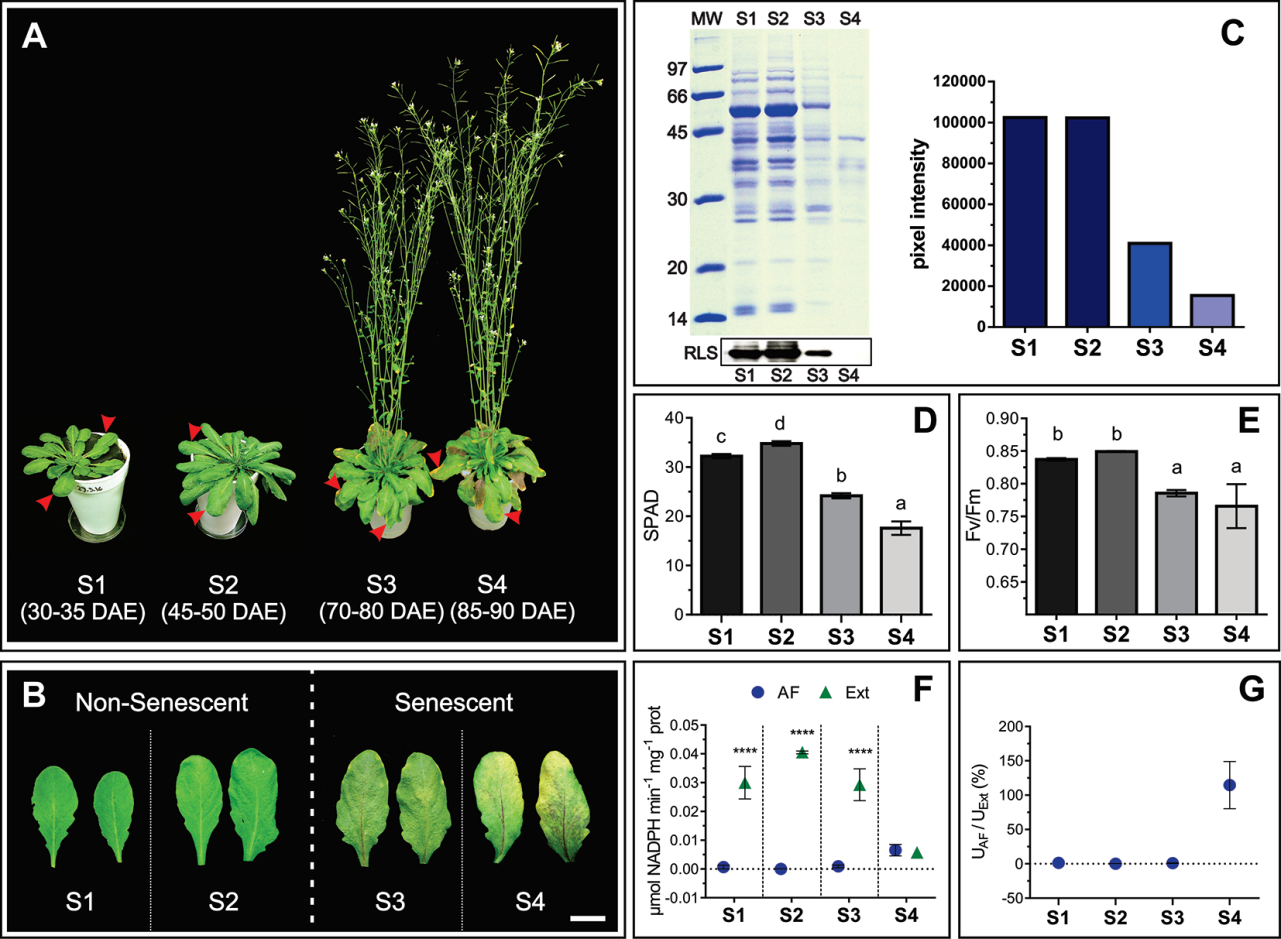
Physiological characteristics of S1, S2, S3, and S4 leaves. **(A)** Leaf stages, marked with red arrows, DAE: Days after emergency. **(B)** Mature, non-senescent leaves (S1 and S2) and senescent leaves (S3 and S4), scale bar 2 cm. **(C)** Leaf protein content: Representative SDS-PAGE showing a total protein profile loaded per unit leaf area, and immunodetection of Rubisco Large Subunit (RLS), in the left upper and lower panel, respectively. Relative leaf protein content, considering S1 as maximum, was estimated by densitometric analysis of the SDS-PAGE, and it is shown on the right panel. **(D)** Chlorophyll content per unit leaf area expressed in SPAD units. **(E)** Maximum quantum yield of Photosystem II (Fv/Fm). **(F)** Enzymatic activity (U) of the cytosolic enzyme G6PDH in apoplastic fluid (AF) and total leaf extracts (Ext) expressed on the basis of total protein (prot) content. **(G)** Percentage of G6PDH activity in AF as a function of G6PDH activity in Ext. Values in the graphs show the average ± standard error, SEM, of independent measurements. Data were normally distributed. SPAD readings were taken in 10 to 15 different leaves for each stage, two readings per leaf blade. Fv/Fm values are the average of 16 leaves (S1), 30 leaves (S2 and S3), and six different S4 leaves, one reading per leaf blade. G6PDH activity **(D** and **E)** represents the average of biological samples (n), where each FA and Ext biological sample was made up of at least 4 leaves. S1: n = 6 FA and Ext n = 6, S2: n = 4 FA and n = 3 Ext, S3: n = 9 FA and n = 5 Ext, S4: n = 3 FA and 3 Ext. Significant differences were calculated at *P* ≤ 0.05 between the conditions using the Tukey test; letters indicate significant differences between stages. Asterisks indicate significant differences between AF and Ext; ****, *P* < 0.0001, calculated using a two-way ANOVA test.

In order to characterize changes in the apoplast accompanying senescence, leaf developmental stages were defined and apoplast fluids (AFs) were isolated. Four leaf stages were characterized: non-senescent leaves from plants at vegetative (S1) and early reproductive stages (S2), and senescing leaves from plants at mid (S3) and late reproductive stages (S4) (**Figures 1A**, **B**). Chl and protein content per leaf area and Photosystem II maximum photochemical efficiency (Fv/Fm) were determined. S1 and S2 leaves showed no difference in Rubisco and total leaf protein content (**Figure 1C**), PSII integrity, as assessed by Fv/Fm, (**Figure 1E**), and S2 Chl concentration was slightly higher than in S1 leaves (**Figure 1D**). S3 and S4 leaves showed clear signs of senescence, i.e. Chl and protein levels and Fv/Fm decreased in S3 and S4 compared to S1 and S2. Particularly, the amount of leaf protein dropped down to 40 and 20% in S3 and S4 respectively, compared to non-senescing S1 and S2 leaves. AF isolation was performed following the vacuum infiltration–centrifugation method (Lohaus et al., 2001; O'Leary et al., 2014) and obtained AF fractions (from here on AWFs) were tested for potential contamination with intracellular compounds. AWFs and whole leaf extracts were assayed for the cytosol marker Glucose 6P Dehydrogenase (G6PDH), which was expressed as G6PDH activity in AWF as a percentage of the whole leaf extract activity (**Figures 1F**, **G**). AWF from S1 to S3 leaves were devoid of detectable G6PDH activity, whereas AWF S4 showed high activity of the cytosolic enzyme, indicating the presence of intracellular compounds in the AWF, probably due to age-related loss of cell membrane integrity (**Figure 1G**). Based on whole-leaf and AWF analysis (**Figures 1A–G**), S3 leaves show clear signs of senescence while still retaining cell integrity, whereas S4 leaves would represent the culmination of the remobilization process followed by membrane permeabilization and cell death. S1 and S2 showed no difference in senescence-related parameters; therefore S2 was selected as non senescent leaf stage to compare its apoplastic space and AF with that of senescing S3 leaves. Both S2 and S3 leaves belong to plants at reproductive stage and that would avoid or minimize any variation in the apoplast related to different phases of plant development.

### Water Relations of Non Senescing and Senescing Leaves. Relative Water Content and Water Potential in S2 and S3 Leaf Stages

**Figure 2:**
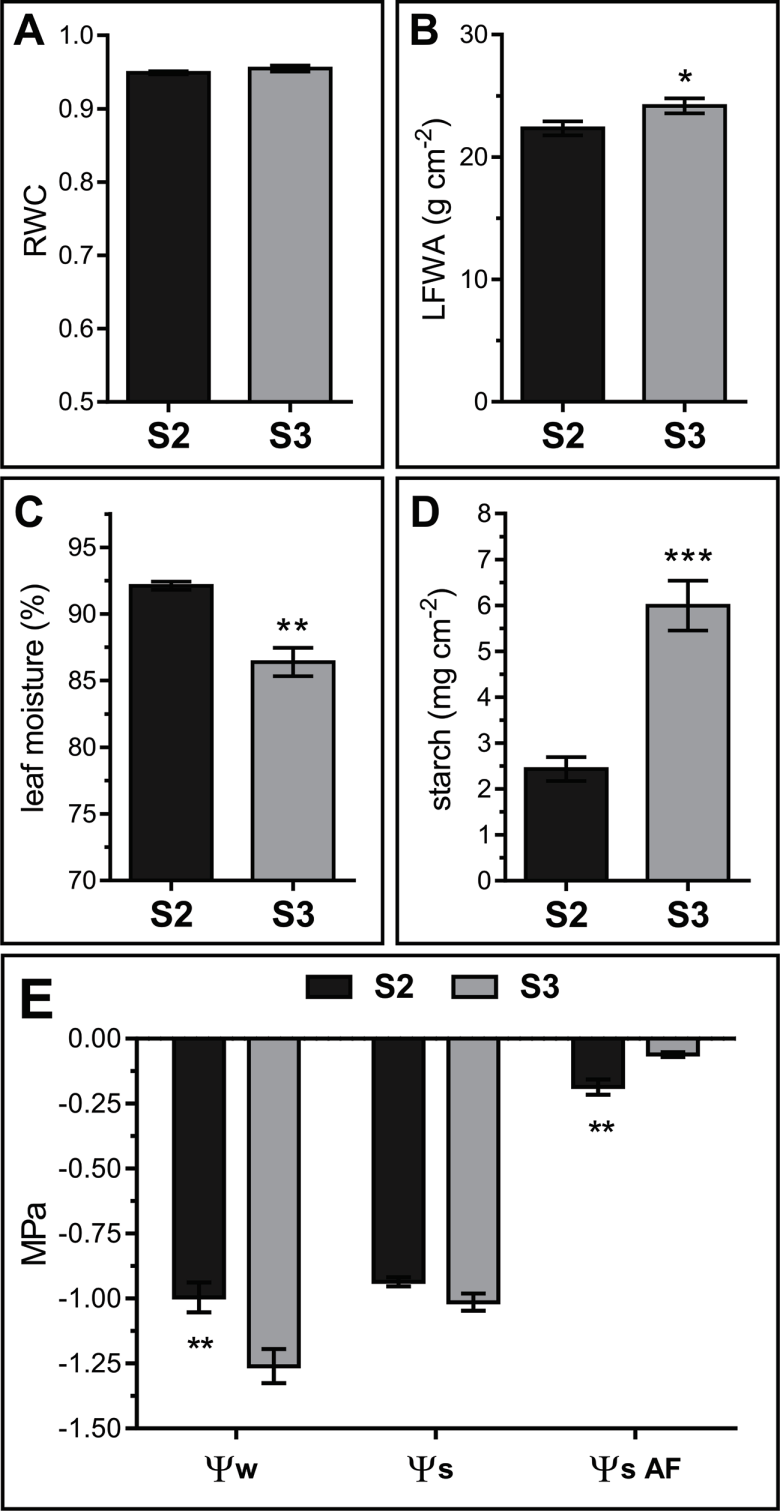
Water relations in S2 and S3 leaves. **(A)** Relative water content (RWC). (**B**) Leaf fresh weight per unit of leaf area (LFWA). **(C)** Leaf water content (LWC), expressed as percentage of water per leaf FW. **(D)** Starch content expressed as mg of glucose per unit leaf area. **(E)** Leaf water potential (Ψ_w_) measured on leaf disks, solute potential of freeze-thaw squeezed leaves (Ψ_s_) and AF solute potential (Ψ_S__AF_). Data represents the average of biological samples (n). **(A)** RWC: n = 6 (S2) and n = 6 (S3), **(B)** LFWA: n = 11 (S2) and n = 13 (S3), **(C)** LWC: n = 6 (S2) and n = 6 (S3), **(D)** starch: n = 5 (S2) and n = 6 (S3), (E) Ψ_w_: n = 13 (S2) and n = 13 (S3), Ψ_s_: n = 4 (S2) and n = 6 (S3), Ψ_S__AF_: n = 4 (S2) and n = 4 (S3). Values were normally distributed and significant differences were calculated between S2 and S3 at *P* ≤ 0.05 using the Student's t test: *, *P* < 0.05; **, *P* < 0.01; ***, *P* < 0.001. Values in the graphs show the average ± SEM.

Leaf water status directly affects the apoplast water content and therefore its metabolite concentration (Tyree, 1976; Andersen et al., 1991; Wardlaw, 2005). Water relations vary throughout *Arabidopsis* leaf development, particularly right after the leaf reaches its maximum extension and starts yellowing (Breeze et al., 2011). Relative water content (RWC), moisture, and water potential (Ψ_w_) were determined in S2 and S3 leaves. RWC remained constant between S2 and S3 stages (S2 RWC = 0.949 ± 0.002 and S3 RWC = 0.955 ± 0.004) (**Figure 2A**). However, percentage leaf water content decreased and leaf fresh weight per area (LFWA) increased in S3 compared to S2 leaves (**Figures 2B**, **C**). A higher LFWA might relate to starch accumulation during senescence (Oda-Yamamizo et al., 2016). The 3-fold increase in starch content detected from S2 to S3 leaves (**Figure 2D**) might explain the higher LFWA in S3 leaves.

Leaf water potential dropped from S2 to S3 (Ψ_w_ = −1.00 ± 0.21 to −1.26 ± 0.25 MPa, respectively), whereas the solute potential remained stable between stages (Ψs S2 = −0.94 ± 0.03 and Ψs S3 = −1.01 ± 0.08) (**Figure 2E**, left and middle parts of the graph). Intriguingly, experimental Ψs values were the same or higher than Ψ_w_ values. Since extracts from frozen-thawed squeezed leaves are composed of cellular, AF and xylem contents, it would be plausible that AF and xylem fluids diluted the cellular sap, leading to higher Ψs than expected based on leaf disk Ψ_w_ values. Yet, a methodological artifact cannot be dismissed since Ψ_w_ was measured in fresh leaf disks with a dew point psychrometer, whereas Ψs was measured in frozen-squeezed leaves using a vapor pressure micro-osmometer.

S2 and S3 AF solute potential (Ψ_S_AF) was estimated measuring AWFs with the vapor pressure microosmometer, and correcting the obtained AWF Ψ_S_ values by the corresponding dilution factor (see *Materials and Methods*, *Leaf AWF Extraction*). Estimated apoplastic Ψ_S_ was significantly higher than leaf Ψs, and differs between leaf stages; Ψ_S_AF values were higher in S3 with respect to S2, thus evidencing a more diluted AF in senescing leaves (**Figure 2E**, right side of the graph).

### Leaf Senescence–Related Changes in the Apoplast Volume, Apoplastic Air-Fluid Ratio, and Cell Wall Properties

To explore potential changes in the extracellular space volume along leaf senescence the apoplastic air (V_air_) and AF (V_AF_) volumes were determined in S2 and S3 leaves (**Table 1**).

**Table 1:** Apoplastic volumes occupied by fluid (AF) and air in S2 and S3 leaves.

	Apoplastic fluid volume (V_AF_)		Apoplastic air volume (V_air_)		F_dil_
	µL cm^−2^	µL g^−1^ LFW	µL cm^−2^	µL g^−1^ LFW	
**S2**	0.49 ± 0.04	21.40 ± 1.57	7.03 ± 0.15	303.50 ± 4.17	18.25 ± 1.38
**S3**	1.34 ± 0.04 ^****^	55.01 ± 1.95 ^****^	7.13 ± 0.11	289.60 ± 2.05 ^***^	6.54 ± 0.22 ^****^

Apoplastic volumes were expressed in μL per gram of leaf fresh weight (LFW) and in μL per unit leaf area. Obtained V_air_ and V_AF_ values were used to estimate AWF dilution factor (F_dil_, see equation in Materials and Methods). Data were normally distributed and significant differences were calculated at *P* ≤ 0.05 between leaf stages using the Student's t test. Each value represents the mean of 35 to 55 biological samples, n, where each sample was made up of at least four leaves. Asterisks indicate significant differences: ***, *P* < 0.001; ****, *P* < 0.0001.

Volume determinations were performed with the Indigo carmine, IC, technique (Husted and Schjoerring, 1995; Lohaus et al., 2001) in multiple independent assays. Determined V_air_ and V_AF_ values were similar to those reported for other species, in the range between 40 and 200 µL per gr of leaf FW (Dietz, 1997; Lohaus et al., 2001; Nouchi et al., 2012). The V_air_ remained stable between S2 and S3 stages, whereas V_air_ was shown to increase with age in *Brassica napus* and *Vicia faba* (Husted and Schjoerring, 1995; Lohaus et al., 2001). When expressed on a leaf fresh weight, LFW, basis there was a slight decrease of 4.6% V_air_ in S3 compared to S2 (303.50 ± 4.17 μL g^−1^ LFW in S2 and 289.60 ± 2.05 μL g^−1^ LFW in S3) whereas when the V_air_ was expressed per unit leaf area there was no significant difference between S2 and S3. On the other hand, V_AF_ increased significantly as leaves aged. S3 leaves contained 157% more AF than S2 leaves (21.40 ± 1.57 μL g^−1^ LFW in S2 and 55.01 ± 1.95 μL g^−1^ LFW in S3). When V_AF_ was expressed per unit leaf area S3 leaves showed a 173% increase compared to S2 (0.49 ± 0.04 μL cm^−2^ and 1.34 ± 0.04 μL cm^−2^ in S2 and S3, respectively). The higher fresh weight per unit area in S3 with respect to S2 leaves (**Figure 2B**) might explain the differences observed when apoplast volumes (V_AF_ or V_air_) are expressed on a leaf area or on a LFW basis. The increased V_AF_ in S3 leaves is consistent with their higher Ψ_S_AF (**Figure 2G**). An age-related increase in V_AF_ was also observed in leaves of *B. napus* and *V. faba* (Husted and Schjoerring, 1995; Lohaus et al., 2001), whereas V_AF_ was reported to decrease during rice leaf senescence (Nouchi et al., 2012). An increase in V_AF_ has also been observed in plants under different types of stress (Rascio et al., 1992; Tetlow and Farrar, 1993; Lohaus et al., 2001; Hamouda et al., 2016).

**Figure 3:**
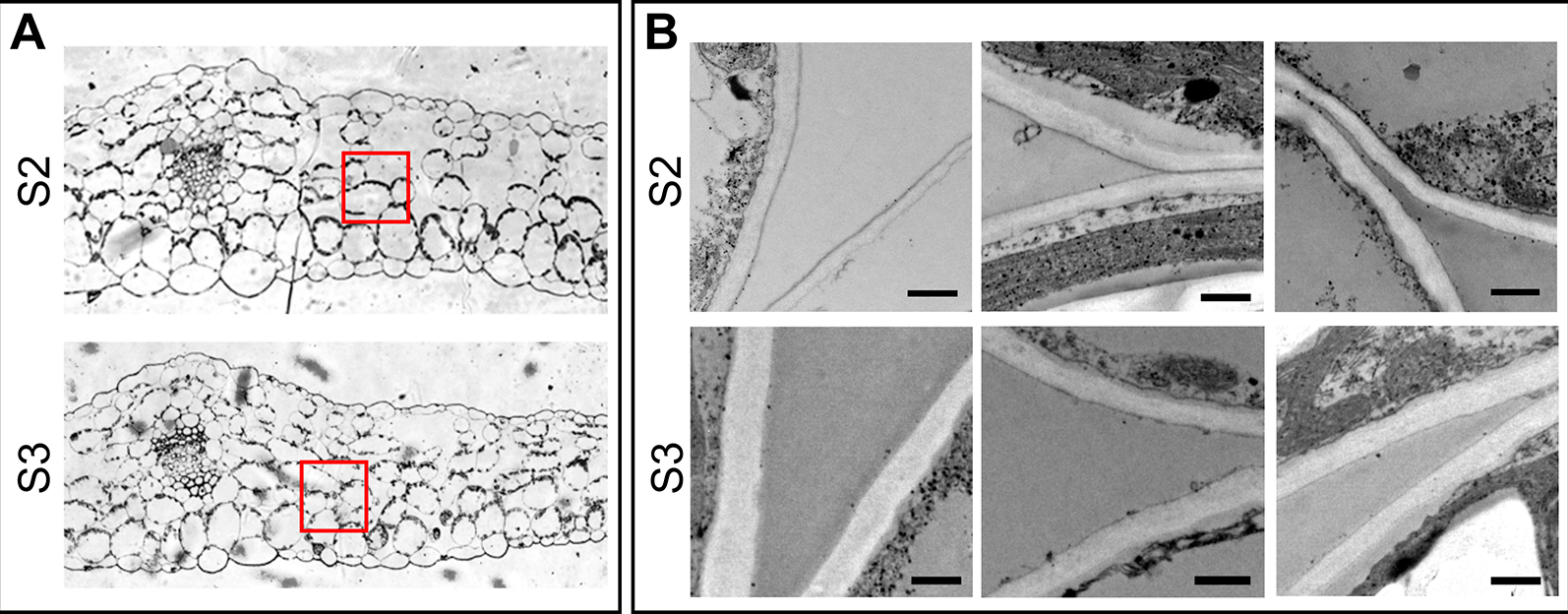
Cell wall thickness of spongy mesophyll cells. **(A)** Transverse section of the middle part of the leaf, showing the areas examined for cell wall thickness. **(B)** Electron microscopy of the selected areas in panel **A**. Upper panel: S2 leaves, lower panel: S3 leaves. Scale bar 500 nm. Cell wall thickness was examined in different cells of at least three different leaves of each Stage. Measurements were taken at different points on the images, the number of total measurements for S2 and S3 were: n = 639 and n = 739, respectively. Obtained values were analyzed by one-way ANOVA, and Univariate Tests of Significance: n (S2 = 639, S3 = 739), mean ± SEM (S2 = 247.9 ± 3.0 nm, S3 = 278.8 ± 2.9), F = 63.02, *P* ≤ 0.0001.

It has been proposed that cell wall mass and structure, particularly the type and content of pectin polymers that affect the water retention potential of the cell walls (Levesque-Tremblay et al., 2015) influence the V_AF_. (Boyer, 1967; Tyree, 1976). We surmised that the steep increase in V_AF_ in S3 leaves would be due to differences in the cell wall properties between leaf stages. To test this hypothesis, the amount of cell wall and pectins, quantified as AIR without starch, and uronic acid concentration, respectively, as well as cell wall thickness were compared between S2 and S3 leaves. Cell wall and pectin content did not differ significantly between S2 and S3, even though there was a slight increase in the pectin content in S3 leaves (**Table 2**). It has been demonstrated that cell-wall components continue to be deposited into the wall even after the cell no longer expands leading to an increase in cell wall thickness (Wei and Lintilhac, 2007), however the cell wall content is similar in S2 and S3. Interestingly, senescing leaves showed slightly thicker spongy mesophyll cell walls compared to those of non-senescing leaves (247.9 ± 3.0 and 278.8 ± 2.9 nm in S2 and S3, respectively) (**Figure 3**). Water retained inside the wall might lead to increased thickness (Forouzesh et al., 2013). Also, modifications such as methylesterifications and ion cross-links directly affect the water binding capacity of pectins, and these changes occur in response to abiotic stress and along leaf development (Levesque-Tremblay et al., 2015). Forouzesh et al. (2013) showed that cell wall thickness increases with plant age, and the oldest leaves have thinner walls compared to the newer ones. The results presented here can be reconciled with those of Forouzesh et al. (2013) since these authors examined cell wall thickness in young and older leaves in the plant, whereas the present study compares non-senescing and senescing leaves that represent the same cohort at two different times of plant development.

**Table 2:** Cell wall and pectin content in S2 and S3 leaves.

Leaf	Cell wall		Pectin	
	mg cm^−2^	mg g^−1^ LFW	mg cm^−2^	mg g^−1^ LFW
**S2**	1.09 ± 0.02	48.16 ± 1.00	0.06 ± 0.00	5.06 ± 0.24
**S3**	1.20 ± 0.09	48.86 ± 2.88	0.08 ± 0.01	6.65 ± 0.54

Values were expressed in mg per gram of leaf fresh weight (LFW) and in mg per unit leaf area. The amount of pectin was expressed as mg of uronic acids. Mean values ± SEM are given. Data were normally distributed and significant differences were calculated at *P* ≤ 0.05 between the stages using the Student's t-test. Each value represents the mean of biological samples, n. S2: n = 3, S3: n = 4, where each sample was made up of a group of S2 or S3 leaves.

### Apoplastic Alkalinization Accompanies Developmental Leaf Senescence

**Figure 4:**
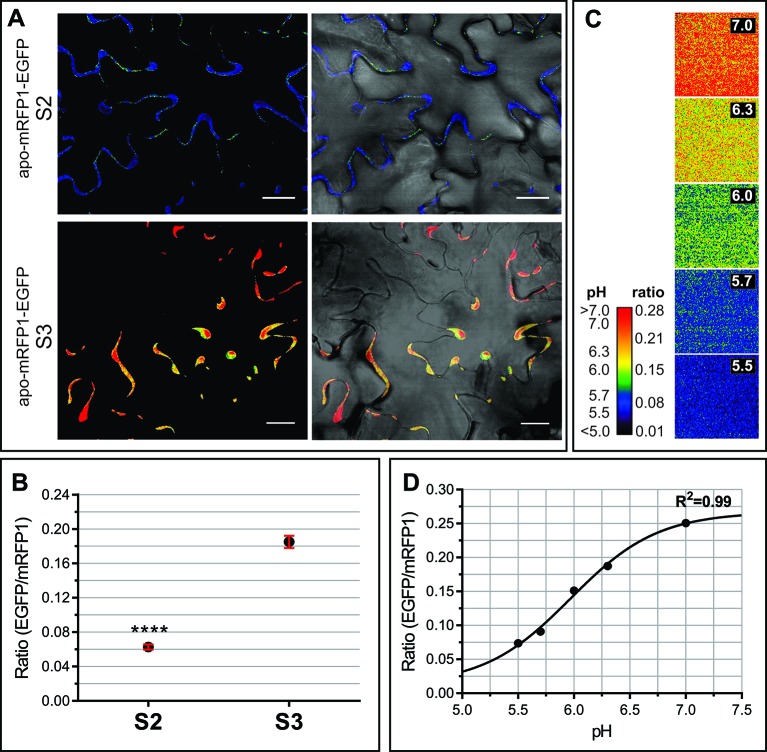
Apoplastic pH of S2 and S3 leaves. *In vivo* analysis of apoplastic pH using the *Arabidopsis* transgenic line apo-mRFP1-EGFP (Gjetting et al., 2012). The pH was estimated according to the fluorescence intensity ratio EGFP/mRFP for each region of interest (ROI). **(A)** Left panels show representative ratio images of the leaf epidermis in S2 and S3 leaves, right panels show the merge of ratio images with their corresponding light field. **(B)** EGFP/mRFP ratio of leaf S2 and S3 epidermal cells. The values shown correspond to the mean ± SEM of 39 and 119 ROIs from S2 and S3 leaves, respectively, taken from at least three different leaves for each stage. A Student t test (*P* ≤ 0.05) was performed, asterisks indicate significant differences: ****, *P* < 0.0001. **(C**, **D)**. *Ex vivo* calibration of the apo-pHusion sensor. **(C)** Ratio images of AWF incubated at different pH units, used to construct the calibration curve. For visual image presentation of ratio images, a pseudocolor look-up table was designed. **(D)** Calibration curve. PH was estimated according to the intensities of GFP and RFP fluorescence. Confocal data acquisition was performed on a Leica TCS SP5 II confocal laser scanning microscope. Image data were analyzed using the ImageJ software. Scale bar = 20 μm.

Transient oscillations and long term changes in apoplastic pH (pH_Apo_) trigger and/or modulate a myriad of signaling and metabolic pathways (Felle, 2001). Possible fluctuations in the pH_Apo_ between S2 and S3 leaves were evaluated by *in vivo* imaging using the genetically encoded pH sensor Apo-pHusion. Apo-pHusion plants stably express the mRFP1-EGFP tandem targeted to the apoplast (Gjetting et al., 2012). Confocal images were acquired at the center of the leaf blade, from epidermal cells, where the fluorescence signal is strong enough to be quantified (**Figure 4**). The pH for each region of interest (ROI) was estimated based on the ratio between EGFP and mRFP1 fluorescence intensities, according to a calibration curve (**Figures 4C**, **D**). Estimated pH_Apo_ shifted around 0.8 units between S2 and S3, from pH 5.43 ± 0.03 in S2 leaves to 6.26 ± 0.05 in S3 leaves (**Figures 4A**, **B**). These pH_Apo_ values are in the range of pH 5 to 6 expected for leaf pH_Apo_ (Felle, 2001), although absolute pH values may be difficult to estimate as they might be influenced by different factors, i.e. calibration. A pH_Apo_ shift of around 1 unit has been reported associated to electrical waves related to long distance signaling triggered by stress (Felle and Zimmermann, 2007). Different types of stress lead to a rise in pH_Apo_ and in extracellular ABA level, a promoter of leaf senescence (Zhao et al., 2016; Geilfus, 2017). It is controversial whether apoplast alkalinization acts as an upstream signal triggering stress responses, or it is a consequence or secondary effect of the stress response (Geilfus, 2017), this controversy could be extended to the senescence-related scenario. Changes in Apo_pH_ affect the activity of extracellular enzymes and transporters, modulating different metabolic pathways (Hothorn et al., 2010).

The NH_3_ released to the extracellular space during developmental and dark-induced senescence converts into NH_4_^+^, that alkalinizes the apoplast (Mattsson and Schjoerring, 2003). NH_4_^+^ accumulation and pH_Apo_ alkalinization rates correlate with the progress of senescence in tobacco (Wu et al., 2016). A rise in pH_Apo_ leads to the ionization of wall molecules, i.e. pectin carboxyl groups, therefore more molecules of water are able to interact with these moieties, which could lead to an increase of cell wall thickness and V_AF._

### Protein Profile of Apoplastic Fluids. Pathogenesis Related Proteins, PR, Accumulate in the Apoplast of Senescing Leaves

**Figure 5:**
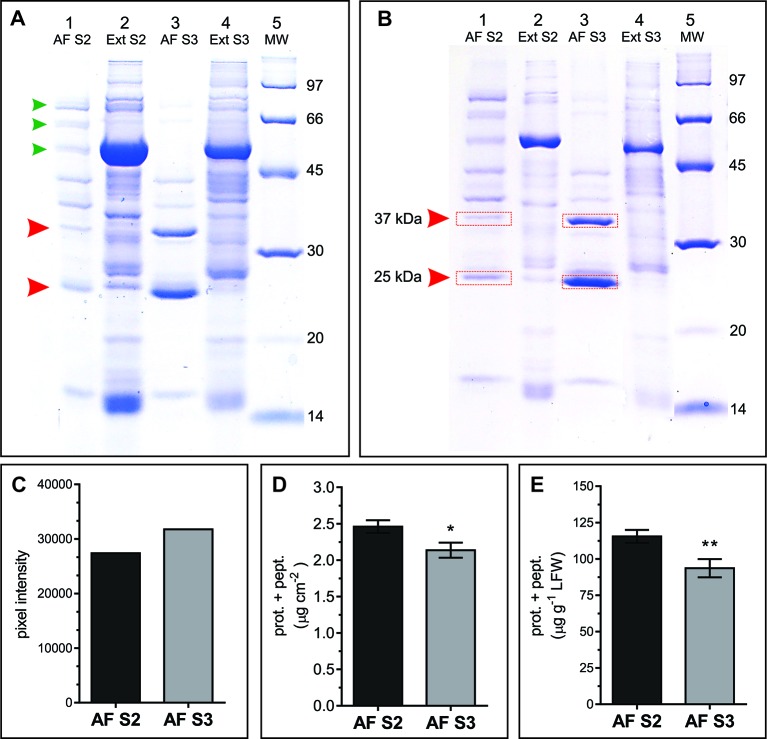
Protein profile of AF S2 and AF S3. **(A**, **B)** Representative SDS-PAGE of total soluble leaf proteins (Ext) and apoplastic fluid (AF). **(A)** Lanes 1 and 3, proteins from 8.64 cm^2^ of leaf; lanes 2 and 4, proteins from 0.196 cm^2^ of leaf. Arrowheads mark bands in AF that differ between S2 and S3. **(B)** Each lane contains ~20 μg of protein. MW: molecular weight markers (masses in kDa). Red rectangles show the bands excised and analyzed by mass spectrometry. **(C)** Densitometric quantification of lanes 1 and 3 from gel A. **(D**, **E)** Total protein and peptide content in AF S2 and AF S3, expressed as µg of proteins and peptides per unit of leaf area **(D)** and as µg of proteins and peptides per gram of leaf fresh weight (LFW), respectively. Mean values ± SEM of biological samples, n. S2 n = 28, S3 n = 30. For each biological sample two technical replicates were taken. Significant differences were calculated at *P* ≤ 0.05 between leaf stages using the Student's t-test: ^*^, *P* < 0.05; ^**^, *P* < 0.01.

AWFs along with the corresponding whole leaf extracts from S2 and S3 leaves were examined in SDS-PAGE gels. In the range of 14 to 100 kDa, the AF protein patterns from S2 and S3 leaves (AFS2 and AFS3) displayed clear quantitative and qualitative differences (**Figure 5**). The AFS2 profile shows less protein per unit leaf area than the AFS3, and it is composed of several uniformly distributed protein bands. Few bands from 45 to 97 kDa are distinguished in AFS2 but not in AFS3 (**Figure 5A**, small arrowheads). The AFS3 protein profile is characterized by two conspicuous bands, one of apparent molecular mass of 25 kDa and another of 37 kDa, that are not clearly recognizable in AFS2 or in total leaf protein profiles (**Figures 5A**, **B**, big arrowheads). The densitometric analysis of SDS-PAGE gels measured a higher protein content per unit leaf area in AFS3 than in AFS2 (**Figure 5C**) whereas, on the other hand, a colorimetric absorbance assay (Pierce™ BCA Protein Assay Kit), that detects all molecular weight proteins and peptides, reveals a higher protein and peptide content in AFS2 than inAFS3 either on a leaf area or LFW basis (**Figures 5D**, **E**). The discrepancy between methods might indicate a decline in the relative abundance of low molecular weight proteins (<14 kDa) and peptides in AFS3.

To identify the proteins corresponding to the two main bands detected in AFS3 protein profiles (**Figure 5A**) equal amounts of AFS2 and AFS3 proteins were loaded on an SDS-PAGE gel (**Figure 5B**), and the 25 and 37 kDa bands from S2 and S3 were cut off the gel and eluted for mass spectrometry analysis.

Peptide sequences identified different proteins in each band. According to the total number of peptide sequences for each identified protein (#PSM), the most abundant proteins were Pathogenesis-related protein 5 (PR5, At1g75040) in the 25 kDa band and endo-1,3 Glucan-beta-glucosidase, acidic isoform (PR2, At3g57260) in the 37 kDa band. Other less represented proteins co-migrating with PR2 and PR5 in the SDS-PAGE were also identified (**Supplemental Tables S1** and **S2**). Both PR2 and PR5 contain an N-terminal signal peptide (SP) for secretion, and PR5 was previously detected in the N-glycosylated sub-proteome of *Arabidopsis* mature stems (Wei et al., 2015). According to sequence homology, PR5 is a thaumatin-like protein (TLP) (Uknes et al., 1992). TLP have antifungal and antifreeze properties and accumulate in response to stress (Velazhahan et al., 1999). PR2 is a cell wall β-1,3-glucanase that hydrolyses the β-1,3 glucosidic bonds of β-1,3-glucans (callose) (Uknes et al., 1992). Besides being found in plant cells, β-1,3-glucans constitute the main structural components of fungal cell walls, hence the antifungal properties of PR2 (Doxey et al., 2007). *PR5* and *PR2* expression is associated with the hypersensitive response (HR), and systemic acquired resistance (SAR) (Geraats et al., 2007; Mishina and Zeier, 2007), and are widely considered hallmark genes for Salicylic acid (SA)–mediated defense and PCD (Uknes et al., 1992; Van Loon and Van Strien, 1999; Sävenstrand et al., 2004; Seo et al., 2008). However, *PR2 and PR5,* along with other *PR* genes are also induced in a SA partially independent manner, for instance, as part of signaling pathways mediated by the protein Di19 in *Arabidopsis* under drought-stress (Liu et al., 2013) and by the plasma membrane-localized, dark induced senescence associated Receptor-Like Kinase, OsSIK2, in rice (Chen et al., 2013).

*PR2* and *PR5* genes are up regulated during leaf senescence (Winter et al., 2007). *PR5* expression is induced by the senescence-age related transcription factor WRKY75 in a SA mediated pathway (Guo et al., 2017). WRKY75 also represses catalase activity, a down regulator of *PR2* expression (Jing et al., 2009).

**Figure 6:**
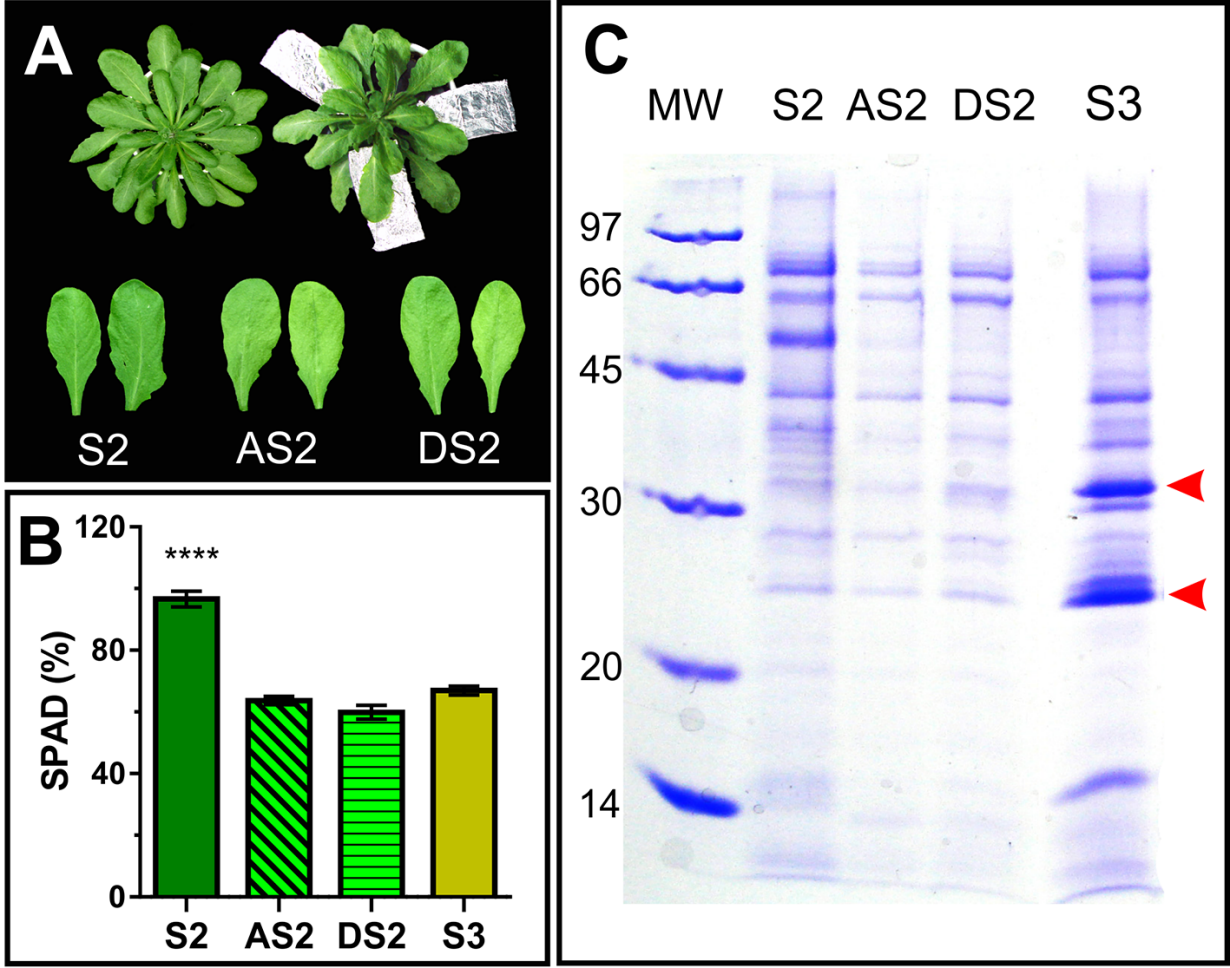
Dark induced senescence of S2 leaves. **(A)** Attached S2 leaves (AS2) were wrapped in aluminum foil, and detached S2 leaves (DS2) were placed on moist filter paper in dark boxes, until their chlorophyll content reached S3 values (**Figure 1**). **(B)** Leaf chlorophyll content was measured non-destructively with the SPAD meter in different leaves, each of them represents a biological sample (n). S2: n = 29, AS2: n = 22, DS2: n = 5, S3: n = 18. For each biological sample, two SPAD readings were taken. Asterisks represent statistical differences between S2 vs. AS2, DS2 and S3 SPAD values (Dunnett's multiple comparisons test). **(C)** SDS-PAGE of AF from S2, dark induced attached and detached S2 (AS2 and DS2, respectively) and S3 leaves, compared per leaf area. Arrowheads show AF S3- associated bands.

To dissect whether the accumulation pattern of PR2 and PR5 in the apoplast, is associated to the senescence syndrome or age-related, S2 leaves were induced to senesce in the dark for further AF evaluation. To this end, attached and detached S2 leaves where exposed to dark until their Chl content dropped down to S3 leaf values (**Figures 6A**, **B**). Dark-induced senescent S2 leaves attached and detached from the plant show lower AF protein content than that of control S2 leaves, and no accumulation of PR2 and PR5 (**Figure 6C**), suggesting that PR2 and PR5 proteins accumulate in the apoplast as part of developmental senescence, but not when senescence is imposed by dark incubation.

### Apoplastic Fluid Proteome Dynamics During Developmental Leaf Senescence

For a more comprehensive repertoire of senescence related AF proteins, AWFs of S2 and S3 leaves were compared by shotgun proteomics. As a result, 212 proteins were identified, 183 of them showed a significant change (at least twofold difference between AFS2 and AFS3), and were considered AF senescence-related proteins, AFSP. Around 13% (24 proteins) of AFSP increased in the apoplast fluid of senescent leaves, S3 (**Table 3**), whereas 87% (159 proteins) of AFSP decreased during senescence (**Supplemental Table S3**).

**Table 3:** AF proteins with two fold increased levels during leaf senescence.

Protein	Protein ID	Gen ID	MW (kDa)	SL SUBA4	Biological process Gene Ontology	Biotic (B)/(A) abiotic associated	gene expression during senescence	SP	N-g.s.	CL	Fold change
Probable LRR receptor-like protein kinase	C0LGG6	At1g51890	98.5	**Ap, M, PM,** N, ER, G	Protein phosphorylation, defense response to bacterium	B	↑	Yes	Yes	**	
PLC-like phosphodiesterases superfamily protein.	F4JQJ7	At4g36945	44.3	**Ap, PM,** ER, N, G, V	Lipid metabolism	B Chakraborty et al. (2016)	No inf.	Yes	Yes	***	2.26
Endochitinase At2g43580	O24598	At2g43580	28.8	**Ap**, ER, G, V	Cell wall macromolecule catabolic process, response to fungus	B	─	Yes	Yes	***	4.75
Endochitinase (CHI, LSC222)	O24603	At2g43570	29.8	**Ap, Mit, PM**, ER, G, V	Cell wall macromolecule catabolic process, leaf senescence, response to virus, systemic acquired resistance	B	↑	Yes	Yes	****	4.12
Pathogenesis-related protein 5 (PR5)	P28493	At1g75040	25.2	**Ap, V, Mit,** ER, G, Cyt	Regulation of anthocyanin biosynthetic process, response to cadmium ion, response to UV-B, response to biotic stimuli, response to virus, systemic acquired resistance	B	↑	Yes	Yes	****	2.42
Glucan endo-1,3-beta-glucosidase, acidic isoform (PR2, BGL2)	P33157	At3g57260	37.3	**Ap, PM, ER, V,** G, Cyt, Chl	Carbohydrate metabolic process, response to cold, systemic acquired resistance, response to biotic stimuli	B-A	↑	Yes	Yes	****	4.06
Ribonuclease 1 (RNS1)	P42813	At2g02990	25.4	**Ap, PM**, ER, V, G	Aging, anthocyanin-containing compound biosynthetic process, cellular response to phosphate starvation, response to wounding	B-A Lebrasseur et al. (2002)	↑	Yes	Yes	****	6.44
Hevein-like preproprotein (HEL, PR4)	P43082	At3g04720	22.9	**Ap**, ER, G, V, Chl	Defense response to bacteria and fungi, killing of cells of other organism, response to ethylene, response to salicylic acid, response to herbivore, response to salt stress, response to virus, systemic acquired resistance	B	↑	Yes	No	***	2.83
Peroxidase 58 (PRX58)	P59120	At5g19880	35.4	**Ap**, Cyt, ER, G, PM, Chl	H_2_O_2_ catabolic process, oxidation-reduction, response to ethylene, response to oxidative stress, response to pathogens	B	↑	Yes	Yes	**	
Germin-like protein subfamily 1 member 13 (GLP6)	P92997	At5g39100	24.1	**Ap**, Cyt, ER, G, Mit, N, Chl	Unknown	B Fuller et al. (2007)	↑	Yes	Yes	**	
Peroxidase 53 (PRX53)	Q42578	At5g06720	35.0	**Ap**, **G**, Cyt, ER, N, Chl, V	H_2_O_2_ catabolic process, oxidation-reduction, defense response to nematodes, response to oxidative stress, flower development	B	─	Yes	Yes	****	4.81
Metalloendoproteinase 3-MMP (At3-MMP)	Q5XF51	At1g24140	43.0	**Ap**, **PM**, **M**, G, ER, Mit, PX, Chl	Proteolysis, stress response	B-A	↑	Yes	Yes	**	
Alpha-amylase 1 (AMY1)	Q8VZ56	At4g25000	47.3	**Ap**, Cyt, ER, G, Chl	Carbohydrate metabolic process, response to ABA, response to gibberellins, stress response	B-A	↑	Yes	Yes	***	2.78
Probable glucan endo-1,3-beta-glucosidase	Q8VZJ2	At4g16260	37.7	**Ap**, **T**, **PM**, ER, G, Chl	Carbohydrate metabolic process, defense response to fungus, defense response to nematodes, response to salt stress	B-A	↑	Yes	No	***	
Peroxidase 54 (PRX54)	Q9FG34	At5g06730	37.3	**Ap, T**, **V**, ER, Cyt, G, N, PM, Chl	H_2_O_2_ catabolic process, oxidation-reduction, response to oxidative stress	B-A Zhao et al. (2007)	↑	Yes	Yes	***	5.06
Peroxidase 52 (PRX52)	Q9FLC0	At5g05340	34.2	**Ap**,**Cyt, G**, ER, N,	H_2_O_2_ catabolic process, lignin biosynthetic process, oxidation-reduction, response to oxidative stress, xylem development	B Chakraborty et al. (2016)	↑	Yes	Yes	**	
Early nodulin-like protein 1 (ENoDL1)	Q9FN39	At5g53870	38.4	**Ap**, **PM**, **M**, ER, G, N, V	Electron transport chain, stress response	A Rizhsky et al. (2004)	↑	Yes	Yes	**	
Lectin-like protein LEC (LEC)	Q9LJR2	At3g15356	29.7	**Ap, Mit, PM, Chl**, G, ER, Cyt, N	Response to chitin, response to ethylene, response to jasmonic acid, defense response to fungus, ethylene-activated signaling pathway, response to wounding	B	No inf.	Yes	Yes	**	2.93
Lectin-like protein	Q9LK72	At3g16530	30.5	**Ap**, **N, PM,** ER, Cyt, G	Response to oomycetes, response to chitin	B	─	Yes	Yes	***	5.20
Non-specific lipid-transfer protein 4 (LTP4)	Q9LLR6	At5g59310	11.4	**Ap**, **M**, ER, G, Mit, Chl	Lipid transport, response to ABA, response to salt stress, water deprivation, pathogen	B-A	↑	Yes	No	**	
Cysteine protease-like protein	Q9SG15	At3g49340	37.7	**Ap**, ER, Cyt, G, Chl, V	Proteolysis	B Little et al. (2007)	↑	Yes	Yes	**	4.66
Peroxidase 34 (PRX34)	Q9SMU8	At3g49120	38.8	**Ap, G, T, V**, ER, Mit, N, PM, Chl	Defense response to bacterium, defense response to fungus, H2O2 catabolic process, oxidation-reduction, response to cytokinin, response to light stimuli, response to oxidative stress, unidimensional cell growth	B –A	↑	Yes	Yes	***	4.03
Defensin-like protein 195 (ATTI-1)	Q42328	At2g43510	9.9	**Ap**, G, Cyt, V, ER	Defense response, defense response to fungus, killing of cells of other organism	B	↑	Yes	No	***	2.36
Endochitinase EP3 (EP3)	Q9M2U5	At3g54420	29.4	**Ap**, G, Chl, V, ER	Cell wall macromolecule catabolic process, chitin catabolic process, defense response, hypersensitive response, polysaccharide catabolic process, response to bacterium, response to wounding, somatic embryogenesis	B-A	↑	Yes	Yes	**	2.00

Four samples (biological replicates) for each leaf stage were analyzed. A confidence level (CL) was assigned to each protein based on the number of biological replicates in which it appears: **, present in two biological replicas; ***, present in three biological replicas and ****, present in the four biological replicas. Rows highlighted in gray: proteins detected only in AF S3.

Gene expression patterns associated with senescence (column “gene expression during senescence”) were examined using the eFP Browser software (Winter et al., 2007).

Subcellular locations and protein functions were determined using the SUBA4 (Hooper et al., 2016) and Gene Ontology (Ashburner et al., 2000; Consortium, 2016) databases. In bold, manually-assigned locations and in normal font, locations that were inferred from electronic annotation (IEA) or predicted. Biotic (B) or abiotic (A) stress related function were assigned according to Gene Ontology, or otherwise experimental data from literature. Presence of SP was determined by the SignalP 4.1 software (Petersen et al., 2011), and presence of potential N-glycosylation sites was determined with the ScanProsite tool (De Castro et al., 2006).

ID, identification; SL, subcellular localization; SP, signal peptide; N-g.s, potential N-glycosylation sites; CL, confidence level; M, membrane; PM, plasma membrane; G, Golgi; N, Nucleo; Cyt, cytoplasm; Chl, chloroplast; Mit, mitochondrion; Ap, apoplast; ER, endoplasmic reticulum; PX, peroxisome; V, vacuole; T, tonoplast; EV, extracellular vesicles (Rutter and Innes, 2017)

**Figure 7:**
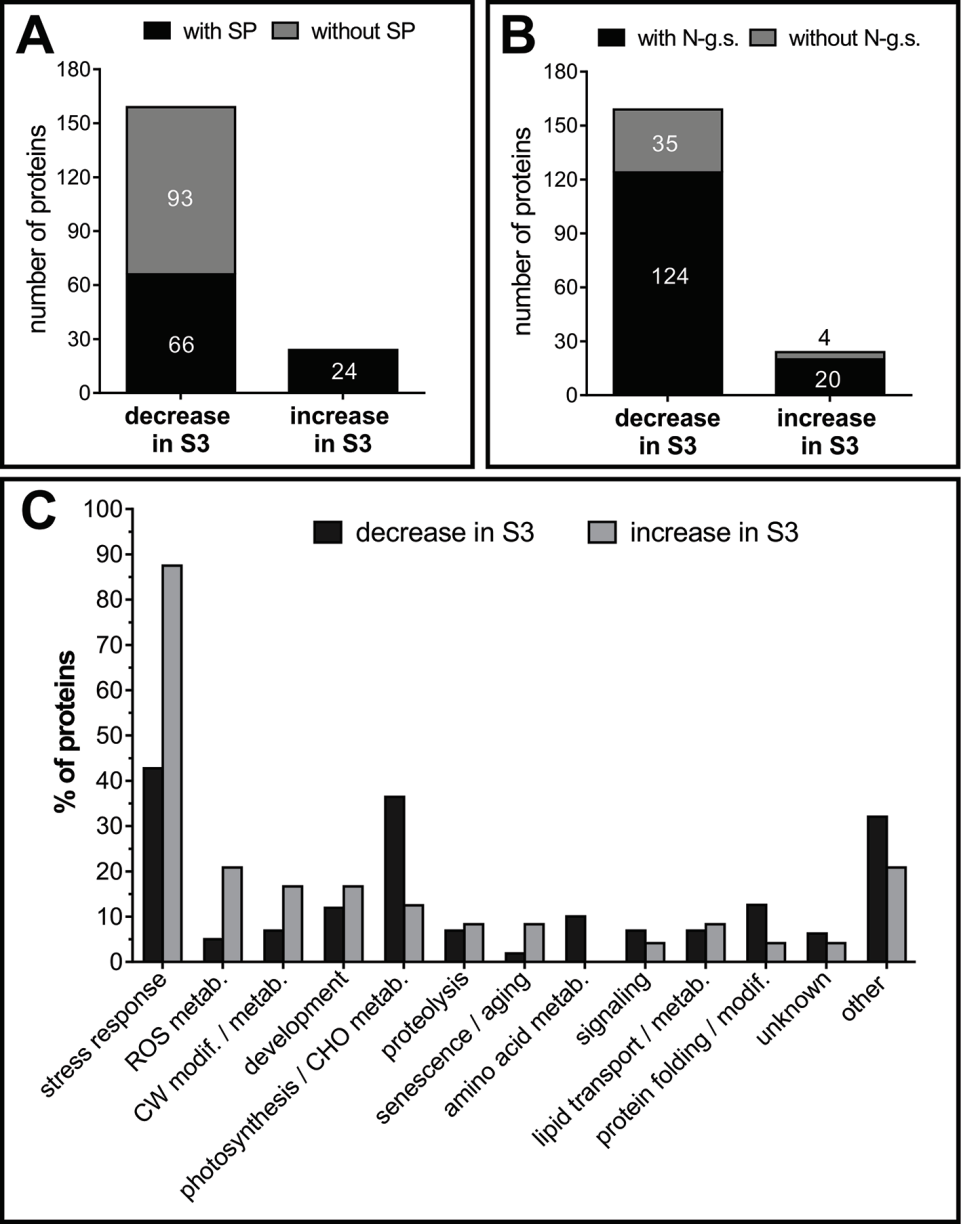
Classification of AF proteins. Presence of signal peptide, SP, **(A)** and potential glycosylation sites **(B)**. Numbers inside and outside the bars indicate the number of proteins. **(C)** Functional classification of AF proteins. Proteins with several known functions were assigned to more than one functional group. Values refer to the percentage of each functional group within the group of proteins that decrease in S3 and within the group of proteins that increase in S3. SP, signal peptide; N-g.s, potential N-glycosylation sites; CW, cell wall; ROS, reactive oxygen species; modif., modification; metab, metabolism; CHO, carbohydrate.

Approximately half of the identified AFSP proteins lack of N-terminal signal peptide (SP), as described in extracellular proteomes before (Agrawal et al., 2010). All the 24 proteins that increased in AFS3 contain SP, whereas only 41.5% of the proteins that decreased in AFS3 contain SP (**Figure 7A**), suggesting that unconventional protein secretion mechanisms that locate leaderless secreted proteins to the apoplast might be less active during leaf senescence. N-glycosylations are posttranslational modifications required for proper folding, transport and/or function of secreted proteins (Song et al., 2013), and the extent and type of N-glycan structure varies as leaf ages (Elbers et al., 2001). Potential glycosylation sites are slightly more represented in proteins that increased in AFS3 compared to those that decline during senescence (**Figure 7B**).

AFSPs were classified into thirteen functional classes (**Figure 7C**), those proteins with several known functions were assigned to more than one functional group. AFS3 proteins relate to the main stress-response group, whereas AFS2 proteins relate mainly to two groups: stress-response and photosynthesis/carbohydrate metabolism.

### Proteins That Accumulate in the Apoplast of Senescent Leaves

PR2 and PR5 were identified among the proteins that accumulate in AFS3. Other 14 proteins significantly accumulate in AFS3 with respect to AFS2: peroxidases: PRX34 (At3g49120), PRX53 (At5g06720), and PRX54 (At5g06730), lectin-like proteins, LLP, (At3g16530 and At3g15356), a cysteine protease (CP1A-5, At3g49340), a serine protease inhibitor (At2g43510), a phosphodiesterase At4g36945, one amylase (AMY1, At4g25000), chitinases (At2g43580, At2g43570, and At3g54420), and ribonucleases (PR4, At3g04720, and RNS1, At2g02990). Eight proteins were specifically detected in AFS3 (not in AFS2): the peroxidases PRX52 (At5g05340) and PRX58 (At5g19880), one lipid transfer protein LTP4 (At5g59310), a serine/threonine kinase (At1g51890), a nodulin-like protein (At5g53870), one glucanase (At4g16260), a germin-like protein (GLP6, At5g39100), and the metallo-protease (3-MMP, At1g24140). According to the information retrieved from the gene expression analysis *platform* EFP-Browser (Winter et al., 2007), all the identified AFS3 corresponding genes are up-regulated in senescing leaves, with the exception of chitinase At2g43580, whose expression remains unchanged.

Interestingly, all the AFS3 identified proteins have been related to stress responses, in particular to biotic stress, with the exception of ENODL1 like protein At5g53870, that was shown to be promoted by drought and heat (Rizhsky et al., 2004); and only few AFS3 proteins have been previously related to leaf senescence. One of them is the amylase AMY1, that locates to the apoplast where it might degrade cell wall associated carbohydrates during *Arabidopsis* senescence (Doyle et al., 2007; Kamranfar et al., 2018), *AMY1* expression is also induced by ABA, heat, and pathogen-related stresses (Doyle et al., 2007). *Arabidopsis amy1* plants show early flowering (Jie et al., 2009). The ribunoclease RNS1 has also been associated to senescence, with a potential role in Pi recycling (Bariola et al., 1994; Bariola et al., 1999). *RNS1* is markedly promoted by Pi deficit and RNS1 protein accumulates in the secretome of Pi starved cells (Tran and Plaxton, 2008). *RNS1* expression is also associated to wounding induced local and systemic signaling (Lebrasseur et al., 2002), and correlates with the accumulation of specific tRNA fragments (Megel et al., 2019). The role of the other identified ribonuclease, PR4, is less known, its expression is associated with SAR (Mishina and Zeier, 2007), and its induced by ethylene and SA (Potter et al., 1993; Camargo-Ramírez et al., 2018).

The five AFS3 peroxidases have been related to pathogen responses. *PRX34* is sharply up regulated during senescence (Winter et al., 2007). PRX34 was identified in the N-glycan secretome of *Arabidopsis* mature stems (Wei et al., 2015) and is also over accumulated under abiotic stress (Mano et al., 2014). *Arabidopsis* knock down plants for PRX34 and its homolog PRX33 (*prx34/prx33)* produce less apoplastic H_2_O_2_ as MAMPs (Microbe-associated molecular patterns) compared to wild type plants (Daudi et al., 2012) and are more sensitive to pathogen attack (O'Brien et al., 2012; Mammarella et al., 2015). PRX34 along with PRX52 accumulate in the leaf apoplast upon *Verticillium longisporum* infection (Floerl et al., 2012). The expression of *PRX54*, is also upregulated in response to fungal infection (Zhao et al., 2007), whereas *AtPRX53* is up regulated in response to nematodes, at their penetration sites, and also in response to wounding and jasmonic acid treatments (Jin et al., 2011). The lipid transfer protein LTP4 accumulates in the AF, along with PR5 and other PR genes, as part of the systemic resistance response induced by *Trichoderma* sp. and *P. syringae* in *Arabidopsis* (Brotman et al., 2012).Two proteases with unknown function accumulate in the AF of S3 leaves. The metallo-protease 3-MMP belongs to the At-MMP family, predicted to be extracellular and related to pathogen responses (Zhao et al., 2017), though *at2-mmp* plants exhibited early senescence and late flowering (Golldack et al., 2002). The cysteine protease At3g49340 is up regulated in response to butterfly egg deposition, causing localized cell death at the site of oviposition (Little et al., 2007). Extracellular cysteine proteases have been shown to mediate defense responses by processing an apoplast immune signaling peptide (Ziemann et al., 2018). At3g49340 might play a similar role in senescence related signaling pathways.

The Germin like protein GLP6, is the only of the identified AFS3 proteins whose expression is down regulated under stress, either abiotic stress, like salt treatment (He et al., 2005), and biotic stress, for example at the galls formed in *Arabidopsis* roots upon infection with parasitic nematodes (Fuller et al., 2007).

### Proteins That Decrease in the Apoplast of Senescent Leaves

The transcript levels of most of the identified proteins that decrease in AFS3 leaves remains unchanged or is down regulated during senescence, except for the aldolase *FBA6* (*At2g36460*), the acid phosphatase AtPAP26 (At5g34850), and the peroxidase *PRX51* (*At4g37530*), that remain constant or are slightly up regulated during leaf senescence (Winter et al., 2007). Based on Gene Ontology (Ashburner et al., 2000) some of the AFSP that decrease in senescing leaves are also stress related, even though no prevalence of biotic stress over abiotic was observed as in ASF3 (**Supplemental Table S3**).

Among ASF2 proteins, the lipid transfer protein LTP6 (At1g55260) was shown to be involved in pathogen elicited responses, and its loss of function increases the susceptibility to penetration of the epidermal cell wall by powdery mildew (Fahlberg et al., 2019). Another LTP (At5g48490), identified only in AFS2, has also been related to SAR responses (Champigny et al., 2013).

The β-glucanase BG3 (At3g57240), the aspartic protease AED (At1g09750, Apoplastic, EDS1-Dependent), and the subtilase SBT 1.7 At5g67360 have also been described as part of pathogen responses, particularly associated to virus infection and to wounding (Golldack et al., 2003; Zavaliev et al., 2013; Breitenbach et al., 2014). The β galactosidase βG60 (At3g13750) was only detected in the AF of S2 leaves; its expression is up regulated by MBF1c (stress-response transcriptional coactivator multiprotein bridging factor) that enhances the tolerance to bacterial infection, heat, and osmotic stress (Suzuki et al., 2005).

Other abiotic stress inducible proteins specifically identified in AFS2 (absent in AFS3), are KIN2 (At5g15970), that responds strongly to ABA, drought and salinity stresses (Kurkela and Borg-Franck, 1992; Zhang et al., 2018), and the pectin methyl esterase At2g46930, that is up regulated under cold stress (Provart et al., 2003).

Few of the identified AFS3 down regulated proteins have been linked to senescence, however, some of them might play specific roles related to senescence. For instance, the chaperone CPN-60 β1 (At1g55490) was previously detected in the apoplast (Bindschedler et al., 2008), and the lack of its activity leads to growth rate reduction and cell death in *Arabidopsis* (Ishikawa et al., 2003) and tobacco (Zabaleta et al., 1994). Anexin AnnAt1 (At1g35720) was detected before in the apoplast (Clark et al., 2005; Bindschedler et al., 2008; Ge et al., 2011), and *at1g35720* plants show early senescence and hypersensitivity to osmotic stress (Lee et al., 2004). Ectopic expression of AnnBj1, the homolog of AnnAt1 in *Brassica juncea*, in tobacco and cotton leads to delayed senescence and increased H_2_O_2_ levels (Jami et al., 2008; Divya et al., 2010).

Some identified enzymes related to ROS (Reactive oxygen species) synthesis and catabolism decrease in AFS3. The superoxide dismutases CSD1 (At1g08830) and CSD2 (At2g28190) are plastidic, but have also been detected in the apoplast (Cheng et al., 2009; Nguyen-Kim et al., 2016). The glyoxylate oxidases GOX1 (At3g14420) and GOX2 (At3g14415) are involved in ROS production. These enzymes lack a SP, but were detected in the apoplast of *Arabidopsis* exposed to oxidative stress (Bindschedler et al., 2008). Double mutant plants *amiRgox1/2* display accelerated leaf senescence (Dellero et al., 2016). AtPAP26 expression is mildly up regulated during senescence (Winter et al., 2007), however its protein level decreases in AFS3. AtPAP26 localized to the apoplast and to the central vacuole is involved in vacuolar Pi recycling and extracellular Pi recruitment (Hurley et al., 2010; Tran et al., 2010). Mutant *atpap26* plants show severe reduction in P recycling, seeds with low P content, and delayed senescence (Robinson et al., 2012).

Interestingly, all of the enzymes related to amino acid metabolism identified in this study decrease in AFS3 (**Supplemental Table S3**). Among them glutamate synthase Fd-GOGAT1 (GLU1, At5g04140) and glutamine synthetase 2 (At5g35630) localize to chloroplast and mitochondria but also to the apoplast (Taira et al., 2004; Bindschedler et al., 2008). Glycine decarboxylase GDCH (At2g35370) also localizes to chloroplast and mitochondria (Kleffmann et al., 2004; Huang et al., 2015), but has not been reported in the apoplast before. GDCH deficiency causes premature senescence in rice (Zhou et al., 2013). The cysteine synthase OASA1 (At4g14880) was previously detected in extracellular vesicles in *Arabidopsis* exposed to oxidative stress (Bindschedler et al., 2008; Rutter and Innes, 2017), and *at4g14880* plants show accelerated leaf senescence (Jing et al., 2002).

The Cyclofilin CYP20-3 (At3g62030) participates in protein folding and cysteine synthesis during stress (Lippuner et al., 1994; Dominguez-Solis et al., 2008; Park et al., 2013), and *cyp20-3* plants senesce faster under oxidative stress (Dominguez-Solis et al., 2008). CYP20-3 is chloroplast located (Lippuner et al., 1994; Hooper et al., 2016), though it was found in the apoplast (Bindschedler et al., 2008), and it would be secreted according to Plant-mPLoc software *in silico* simulations (Chou and Shen, 2010).

## Conclusions

The two most abundant proteins in the apoplast of senescing leaves, PR2 and PR5, along with all the identified apoplastic proteins that increase during senescence, have been previously linked to stress responses.

Leaf senescence-related changes in the secretome, along with variations in the AF volume and pH resemble pathogen attack responses, that in turn involve the induction of many Senescence Associated Genes, SAGs genes (Little et al., 2007; Schippers et al., 2015). PR5 markedly accumulates in the apoplast in response to a variety of biotic and abiotic stresses (Piofczyk et al., 2015; Lambertucci et al., 2019), suggesting a role in a possible universal stress response, or in a crosstalk point where different stress responses converge. It would be plausible that stress-related and senescence programs trigger common mechanisms under conditions requiring recycling. Biotic and abiotic responses are differentially prioritized in a leaf dependent manner in *Arabidopsis* (Berens et al., 2019). Leaves of different ages differentially control responses to stress, allowing for a balance to maintain growth or survival at the organism level (Farber and Mundt, 2017; Berens et al., 2019).

Dark induced-senescence genes are up regulated in *Arabidopsis* infected with tobacco rattle virus, TRV (Fernandez-Calvino et al., 2016), however PR2 and PR5 showed no accumulation in the apoplast of mature healthy leaves (S2) induced to senesce in darkness (**Figure 6**). The specific accumulation of PR2 and PR5 in the apoplast during natural senescence, but not during dark induced senescence suggests that age-related signals, independently of the sink-source status of the leaf, might be priming the apoplast for stress responses in an as yet undamaged leaf. Salicylic Acid, SA, accumulates in an age- related way, and under SA basal levels defense responses are promoted and PR proteins accumulate.

Comparative proteomics of vascular sap reveals a common theme among apoplast fluid, xylem, and phloem sap proteomes collected from stressed plants, that is the accumulation of PR proteins including thaumatin-like proteins, chitinases, and glucanases (Rodríguez-Celma et al., 2016).

The extracellular space might thus represent an up-to-date dismissed scenario where developmental senescence and stress related pathways overlap and integrate.

## Data Availability Statement

This article contains previously unpublished data. The name of the repository and accession number(s) are available at https://figshare.com/projects/FPS_manuscript_476421_Senescence_related_changes_in_leaf_apoplast/71789.

## Author Contributions

MB, JG, and DM designed the experiments. MB and MM performed the experiments. DM and JG wrote the article. MB, JG, and DM discussed the data and revised the article. All authors approved the final manuscript.

## Funding

This work was supported by grant from Agencia nacional de Promoción Científica y Tecnológica, Pres. BID. Pict 1092.

## Conflict of Interest

The authors declare that the research was conducted in the absence of any commercial or financial relationships that could be construed as a potential conflict of interest.

## Abbreviations

AF, apoplastic fluid; AWF, apoplastic wash fluid; AFS2–AFS3, apoplastic fluid protein pattern from S2 and S3 leaves, respectively; AFSP, apoplastic fluid senescence-related protein.

## References

[B1] Agrawal G. K. Jwa N. S. Lebrun M. H. Job D. Rakwal R. (2010). Plant secretome: unlocking secrets of the secreted proteins. Proteomics 10, 799–827. 10.1002/pmic.200900514 19953550

[B2] Andersen M. Jensen C. Losch R. (1991). Derivation of pressure-volume curves by a non-linear regression procedure and determination of apoplastic water. J. Exp. Bot. 42, 159–165. 10.1093/jxb/42.2.159

[B3] Ashburner M. Ball C. A. Blake J. A. Botstein D. Butler H. Cherry J. M. (2000). Gene ontology: tool for the unification of biology. Nat. Genet. 25, 25–29. 10.1038/75556 10802651 PMC3037419

[B4] Bariola P. A. Howard C. J. Taylor C. B. Verburg M. T. Jaglan V. D. Green P. J. (1994). The Arabidopsis ribonuclease gene RNS1 is tightly controlled in response to phosphate limitation. Plant J. 6, 673–685. 10.1046/j.1365-313X.1994.6050673.x 8000425

[B5] Bariola P. A. Macintosh G. C. Green P. J. (1999). Regulation of S-like ribonuclease levels in Arabidopsis. Antisense inhibition of RNS1 or RNS2 elevates anthocyanin accumulation. Plant Physiol. 119, 331–342. 10.1104/pp.119.1.331 9880376 PMC32237

[B6] Berens M. L. Wolinska K. W. Spaepen S. Ziegler J. Nobori T. Nair A. (2019). Balancing trade-offs between biotic and abiotic stress responses through leaf age-dependent variation in stress hormone cross-talk. Proc. Natl. Acad. Sci. 116, 2364–2373. 10.1073/pnas.1817233116 30674663 PMC6369802

[B7] Bindschedler L. V. Palmblad M. Cramer R. (2008). Hydroponic isotope labelling of entire plants (HILEP) for quantitative plant proteomics; an oxidative stress case study. Phytochemistry 69, 1962–1972. 10.1016/j.phytochem.2008.04.007 18538804

[B8] Blumenkrantz N. Asboe-Hansen G. (1973). New method for quantitative determination of uronic acids. Anal. Biochem. 54, 484–489. 10.1016/0003-2697(73)90377-1 4269305

[B9] Boudart G. Jamet E. Rossignol M. Lafitte C. Borderies G. Jauneau A. (2005). Cell wall proteins in apoplastic fluids of Arabidopsis thaliana rosettes: identification by mass spectrometry and bioinformatics. Proteomics 5, 212–221. 10.1002/pmic.200400882 15593128

[B10] Boyer J. (1967). Matric potentials of leaves. Plant Physiol. 42, 213–217. 10.1104/pp.42.2.213 16656497 PMC1086513

[B11] Breeze E. Harrison E. Mchattie S. Hughes L. Hickman R. Hill C. (2011). High-resolution temporal profiling of transcripts during Arabidopsis leaf senescence reveals a distinct chronology of processes and regulation. Plant Cell 23, 873–894. 10.1105/tpc.111.083345 21447789 PMC3082270

[B12] Breitenbach H. H. Wenig M. Wittek F. Jorda L. Maldonado-Alconada A. M. Sarioglu H. (2014). Contrasting Roles of the Apoplastic Aspartyl Protease APOPLASTIC, ENHANCED DISEASE SUSCEPTIBILITY1-DEPENDENT1 and LEGUME LECTIN-LIKE PROTEIN1 in Arabidopsis Systemic Acquired Resistance. Plant Physiol. 165, 791–809. 10.1104/pp.114.239665 24755512 PMC4044859

[B13] Brotman Y. Lisec J. Meret M. Chet I. Willmitzer L. Viterbo A. (2012). Transcript and metabolite analysis of the Trichoderma-induced systemic resistance response to Pseudomonas syringae in Arabidopsis thaliana. Microbiology 158, 139–146. 10.1099/mic.0.052621-0 21852347

[B14] Camargo-Ramírez R. Val-Torregrosa B. San Segundo B. (2018). MiR858-Mediated regulation of flavonoid-specific MYB transcription factor genes controls resistance to pathogen infection in arabidopsis. Plant Cell Physiol. 59, 190–204. 10.1093/pcp/pcx175 29149328

[B15] Carpita N. C. Kanabus J. (1987). Extraction of starch by dimethyl sulfoxide and quantitation by enzymatic assay. Anal. Biochem. 161, 132–139. 10.1016/0003-2697(87)90662-2 3107426

[B16] Chakraborty S. Nascimento R. Zaini P. A. Gouran H. Rao B. J. Goulart L. R. (2016). Sequence/structural analysis of xylem proteome emphasizes pathogenesis-related proteins, chitinases and beta-1, 3-glucanases as key players in grapevine defense against Xylella fastidiosa. PeerJ 4, e2007. 10.7717/peerj2007 27257535 PMC4888286

[B17] Champigny M. J. Isaacs M. Carella P. Faubert J. Fobert P. R. Cameron R. K. (2013). Long distance movement of DIR1 and investigation of the role of DIR1-like during systemic acquired resistance in Arabidopsis. Front. Plant Sci. 4, 230. 10.3389/fpls.2013.00230 23847635 PMC3701462

[B18] Chen L. J. Wuriyanghan H. Zhang Y. Q. Duan K. X. Chen H. W. Li Q. T. (2013). An S-domain receptor-like kinase, OsSIK2, confers abiotic stress tolerance and delays dark-induced leaf senescence in rice. Plant Physiol. 163, 1752–1765. 10.1104/pp.113.224881 24143807 PMC3850199

[B19] Cheng F. Y. Blackburn K. Lin Y. M. Goshe M. B. Williamson J. D. (2009). Absolute protein quantification by LC/MS(E) for global analysis of salicylic acid-induced plant protein secretion responses. J. Proteome Res. 8, 82–93. 10.1021/pr800649s 18998720

[B20] Chou K.-C. Shen H.-B. (2010). Plant-mPLoc: a top-down strategy to augment the power for predicting plant protein subcellular localization. PloS One 5, e11335. 10.1371/journal.pone.0011335 20596258 PMC2893129

[B21] Clark G. B. Lee D. Dauwalder M. Roux S. J. (2005). Immunolocalization and histochemical evidence for the association of two different Arabidopsis annexins with secretion during early seedling growth and development. Planta 220, 621–631. 10.1007/s00425-004-1374-7 15368128

[B22] Coffeen W. C. Wolpert T. J. (2004). Purification and characterization of serine proteases that exhibit caspase-like activity and are associated with programmed cell death in Avena sativa. Plant Cell 16, 857–873. 10.1105/tpc.017947 15020745 PMC412861

[B23] Consortium G. O. (2016). Expansion of the Gene Ontology knowledgebase and resources. Nucleic Acids Res. 45, D331–D338. 10.1093/nar/gkw1108 27899567 PMC5210579

[B24] Dani V. Simon W. J. Duranti M. Croy R. R. (2005). Changes in the tobacco leaf apoplast proteome in response to salt stress. Proteomics 5, 737–745. 10.1002/pmic.200401119 15682462

[B25] Daudi A. Cheng Z. O'brien J. A. Mammarella N. Khan S. Ausubel F. M. (2012). The apoplastic oxidative burst peroxidase in Arabidopsis is a major component of pattern-triggered immunity. Plant Cell 24, 275–287. 10.1105/tpc.111.093039 22247251 PMC3289579

[B26] De Castro E. Sigrist C. J. Gattiker A. Bulliard V. Langendijk-Genevaux P. S. Gasteiger E. (2006). ScanProsite: detection of PROSITE signature matches and ProRule-associated functional and structural residues in proteins. Nucleic Acids Res. 34, W362–W365. 10.1093/nar/gkl124 16845026 PMC1538847

[B27] Dellero Y. Jossier M. Glab N. Oury C. Tcherkez G. Hodges M. (2016). Decreased glycolate oxidase activity leads to altered carbon allocation and leaf senescence after a transfer from high CO2 to ambient air in Arabidopsis thaliana. J. Exp. Bot. 67, 3149–3163. 10.1093/jxb/erw054 26896850

[B28] Delorme V. G. Mccabe P. F. Kim D.-J. Leaver C. J. (2000). A matrix metalloproteinase gene is expressed at the boundary of senescence and programmed cell death in cucumber. Plant Physiol. 123, 917–928. 10.1104/pp.123.3.917 10889240 PMC59054

[B29] Dietz K. J. (1997). “Functions and responses of the leaf apoplast under stress,” in Progress in Botany. Eds. Behnke H. D. Lüttge U. Esser K. Kadereit J. W. Runge M. (Berlin, Heidelberg: Springer), 221–254. 10.1007/978-3-642-60458-4_9

[B30] Divya K. Jami S. Kirti P. (2010). Constitutive expression of mustard annexin, AnnBj1 enhances abiotic stress tolerance and fiber quality in cotton under stress. Plant Mol. Biol. 73, 293–308. 10.1007/s11103-010-9615-6 20148350

[B31] Dominguez-Solis J. R. He Z. Lima A. Ting J. Buchanan B. B. Luan S. (2008). A cyclophilin links redox and light signals to cysteine biosynthesis and stress responses in chloroplasts. Proc. Natl. Acad. Sci. 105, 16386–16391. 10.1073/pnas.0808204105 18845687 PMC2571017

[B32] Doxey A. C. Yaish M. W. Moffatt B. A. Griffith M. Mcconkey B. J. (2007). Functional divergence in the Arabidopsis β-1, 3-glucanase gene family inferred by phylogenetic reconstruction of expression state. Mol. Biol. Evol. 24, 1045–1055. 10.1093/molbev/msm024 17272678

[B33] Doyle E. A. Lane A. M. Sides J. M. Mudgett M. B. Monroe J. D. (2007). An α-amylase (At4g25000) in Arabidopsis leaves is secreted and induced by biotic and abiotic stress. Plant Cell Environ. 30, 388–398. 10.1111/j.1365-3040.2006.01624.x 17324226

[B34] Elbers I. J. Stoopen G. M. Bakker H. Stevens L. H. Bardor M. Molthoff J. W. (2001). Influence of Growth Conditions and Developmental Stage onN-Glycan Heterogeneity of Transgenic Immunoglobulin G and Endogenous Proteins in Tobacco Leaves. Plant Physiol. 126, 1314–1322. 10.1104/pp.126.31314 11457982 PMC116488

[B35] Fahlberg P. Buhot N. Johansson O. N. Andersson M. X. (2019). Involvement of lipid transfer proteins in resistance against a non-host powdery mildew in Arabidopsis thaliana. Mol. Plant Pathol. 20, 69–77. 10.1111/mpp.12740 30102837 PMC6430466

[B36] Farber D. Mundt C. (2017). Effect of plant age and leaf position on susceptibility to wheat stripe rust. Phytopathology 107, 412–417. 10.1094/PHYTO-07-16-0284-R 27898264

[B37] Felle H. H. Zimmermann M. R. (2007). Systemic signalling in barley through action potentials. Planta 226, 203. 10.1007/s00425-006-0458-y 17226028

[B38] Felle H. H. Herrmann A. Hanstein S. Hückelhoven R. Kogel K.-H. (2004). Apoplastic pH signaling in barley leaves attacked by the powdery mildew fungus *Blumeria graminis* f. sp. *hordei* . Mol. Plant-Microbe Interact. 17, 118–123. 10.1094/MPMI.2004.17.1.118 14714875

[B39] Felle H. (2001). pH: signal and messenger in plant cells. Plant Biol. 3, 577–591. 10.1055/s-2001-19372

[B40] Fernandez-Calvino L. Guzman-Benito I. Del Toro F. J. Donaire L. Castro-Sanz A. B. Ruiz-Ferrer V. (2016). Activation of senescence-associated Dark-inducible (DIN) genes during infection contributes to enhanced susceptibility to plant viruses. Mol. Plant Pathol. 17, 3–15. 10.1111/mpp.12257 25787925 PMC6638341

[B41] Floerl S. Majcherczyk A. Possienke M. Feussner K. Tappe H. Gatz C. (2012). Verticillium longisporum infection affects the leaf apoplastic proteome, metabolome, and cell wall properties in Arabidopsis thaliana. PloS One 7, e31435. 10.1371/journal.pone.0031435 22363647 PMC3282744

[B42] Forouzesh E. Goel A. Mackenzie S. A. Turner J. A. (2013). In vivo extraction of Arabidopsis cell turgor pressure using nanoindentation in conjunction with finite element modeling. Plant J. 73, 509–520. 10.1111/tpj.12042 23036157

[B43] Fuller V. L. Lilley C. J. Atkinson H. J. Urwin P. E. (2007). Differential gene expression in Arabidopsis following infection by plant-parasitic nematodes Meloidogyne incognita and Heterodera schachtii. Mol. Plant Pathol. 8, 595–609. 10.1111/j.1364-3703.2007.00416.x 20507524

[B44] Ge W. Song Y. Zhang C. Zhang Y. Burlingame A. L. Guo Y. (2011). Proteomic analyses of apoplastic proteins from germinating Arabidopsis thaliana pollen. Biochim. Biophys. Acta 1814, 1964–1973. 10.1016/j.bbapap.2011.07.013 21798377 PMC3214693

[B45] Geilfus C. M. Mithöfer A. Ludwig-Müller J. Zörb C. Muehling K. H. (2015). Chloride-inducible transient apoplastic alkalinizations induce stomata closure by controlling abscisic acid distribution between leaf apoplast and guard cells in salt-stressed Vicia faba. New Phytol. 208, 803–816. 10.1111/nph.13507 26096890

[B46] Geilfus C.-M. (2017). The pH of the apoplast: dynamic factor with functional impact under stress. Mol. Plant 10, 1371–1386. 10.1016/j.molp.2017.09.018 28987886

[B47] Geraats B. P. Bakker P. A. Linthorst H. J. Hoekstra J. Van Loon L. C. (2007). The enhanced disease susceptibility phenotype of ethylene-insensitive tobacco cannot be counteracted by inducing resistance or application of bacterial antagonists. Physiol. Mol. Plant Pathol. 70, 10. 10.1016/j.pmpp.2007.07.003

[B48] Gjetting S. K. Ytting C. K. Schulz A. Fuglsang A. T. (2012). Live imaging of intra-and extracellular pH in plants using pHusion, a novel genetically encoded biosensor. J. Exp. Bot. 63, 3207–3218. 10.1093/jxb/ers040 22407646 PMC3350929

[B49] Golldack D. Popova O. V. Dietz K.-J. (2002). Mutation of the matrix metalloproteinase At2-MMP inhibits growth and causes late flowering and early senescence in Arabidopsis. J. Biol. Chem. 277, 5541–5547. 10.1074/jbc.M106197200 11726650

[B50] Golldack D. Vera P. Dietz K. J. (2003). Expression of subtilisin-like serine proteases in Arabidopsis thaliana is cell-specific and responds to jasmonic acid and heavy metals with developmental differences. Physiol. Plant 118, 64–73. 10.1034/j.1399-3054.2003.00087.x 12702015

[B51] Goulet C. Goulet C. Goulet M. C. Michaud D. (2010). 2-DE proteome maps for the leaf apoplast of Nicotiana benthamiana. Proteomics 10, 2536–2544. 10.1002/pmic.200900382 20422621

[B52] Grudkowska M. Zagdanska B. (2004). Multifunctional role of plant cysteine proteinases. Acta Biochim. Pol. 51, 609–624. 10.18388/abp.2004_3547 15448724

[B53] Guiamet J. J. Tyystjarvi E. Tyystjarvi T. John I. Kairavuo M. Pichersky E. (2002). Photoinhibition and loss of photosystem II reaction centre proteins during senescence of soybean leaves. Enhancement of photoinhibition by the ‘stay-green' mutation cytG. Physiol. Plant 115, 468–478. 10.1034/j.1399-3054.2002.1150317.x 12081540

[B54] Guo P. Li Z. Huang P. Li B. Fang S. Chu J. (2017). A tripartite amplification loop involving the transcription factor WRKY75, salicylic acid, and reactive oxygen species accelerates leaf senescence. Plant Cell 29, 2854–2870. 10.1105/tpc.17.00438 29061866 PMC5728132

[B55] Gupta R. Lee S. E. Agrawal G. K. Rakwal R. Park S. Wang Y. (2015). Understanding the plant-pathogen interactions in the context of proteomics-generated apoplastic proteins inventory. Front. In Plant Sci. 6, 352. 10.3389/fpls.2015.00352 26082784 PMC4451336

[B56] Hamouda I. Badri M. Mejri M. Cruz C. Siddique K. Hessini K. (2016). Salt tolerance of *Beta macrocarpa* is associated with efficient osmotic adjustment and increased apoplastic water content. Plant Biol. 18, 369–375. 10.1111/plb.12419 26588061

[B57] He X. J. Mu R. L. Cao W. H. Zhang Z. G. Zhang J. S. Chen S. Y. (2005). AtNAC2, a transcription factor downstream of ethylene and auxin signaling pathways, is involved in salt stress response and lateral root development. Plant J. 44, 903–916. 10.1111/j.1365-313X.2005.02575.x 16359384

[B58] Higgins R. Lockwood T. Holley S. Yalamanchili R. Stratmann J. W. (2007). Changes in extracellular pH are neither required nor sufficient for activation of mitogen-activated protein kinases (MAPKs) in response to systemin and fusicoccin in tomato. Planta 225, 1535–1546. 10.1007/s00425-006-0440-8 17109147

[B59] Hooper C. M. Castleden I. R. Tanz S. K. Aryamanesh N. Millar A. H. (2016). SUBA4: the interactive data analysis centre for Arabidopsis subcellular protein locations. Nucleic Acids Res. 45, D1064–D1074. 10.1093/nar/gkw1041 27899614 PMC5210537

[B60] Hoson T. (1998). Apoplast as the site of response to environmental signals. J. Plant Res. 111, 167–177. 10.1007/BF02507163 11541948

[B61] Hothorn M. Van Den Ende W. Lammens W. Rybin V. Scheffzek K. (2010). Structural insights into the pH-controlled targeting of plant cell-wall invertase by a specific inhibitor protein. Proc. Natl. Acad. Sci. 107, 17427–17432. 10.1073/pnas.1004481107 20858733 PMC2951410

[B62] Huang S. Nelson C. J. Li L. Taylor N. L. Ströher E. Petereit J. (2015). INTERMEDIATE CLEAVAGE PEPTIDASE55 modifies enzyme amino termini and alters protein stability in Arabidopsis mitochondria. Plant Physiol. 168, 415–427. 10.1104/pp.15.00300 25862457 PMC4453787

[B63] Hurley B. A. Tran H. T. Marty N. J. Park J. Snedden W. A. Mullen R. T. (2010). The dual-targeted purple acid phosphatase isozyme AtPAP26 is essential for efficient acclimation of Arabidopsis to nutritional phosphate deprivation. Plant Physiol. 153, 1112–1122. 10.1104/pp.110.153270 20348213 PMC2899917

[B64] Husted S. Schjoerring J. K. (1995). Apoplastic pH and Ammonium Concentration in Leaves of *Brassica napus* L. Plant Physiol. 109, 1453–1460. 10.1104/pp.109.41453 12228682 PMC157681

[B65] Ishikawa A. Tanaka H. Nakai M. Asahi T. (2003). Deletion of a chaperonin 60β gene leads to cell death in the Arabidopsis lesion initiation 1 mutant. Plant Cell Physiol. 44, 255–261. 10.1093/pcp/pcg031 12668771

[B66] Jami S. K. Clark G. B. Turlapati S. A. Handley C. Roux S. J. Kirti P. B. (2008). Ectopic expression of an annexin from *Brassica juncea* confers tolerance to abiotic and biotic stress treatments in transgenic tobacco. Plant Physiol. Biochem. 46, 1019–1030. 10.1016/j.plaphy.2008.07.006 18768323

[B67] Jie W. Dashi Y. Xinhong G. Xuanming L. (2009). Arabidopsis AMY1 expressions and early flowering mutant phenotype. BMB Rep. 42, 101–105. 10.5483/BMBRep.2009.42.2.101 19250611

[B68] Jin Y. Ni D.-A. Ruan Y.-L. (2009). Posttranslational elevation of cell wall invertase activity by silencing its inhibitor in tomato delays leaf senescence and increases seed weight and fruit hexose level. Plant Cell 21, 2072–2089. 10.1105/tpc.108.063719 19574437 PMC2729613

[B69] Jin J. Hewezi T. Baum T. J. (2011). Arabidopsis peroxidase AtPRX53 influences cell elongation and susceptibility to Heterodera schachtii. Plant Signal Behav. 6, 1778–1786. 10.4161/psb.6.11.17684 22212122 PMC3329352

[B70] Jing H. C. Sturre M. J. Hille J. Dijkwel P. P. (2002). Arabidopsis onset of leaf death mutants identify a regulatory pathway controlling leaf senescence. Plant J. 32, 51–63. 10.1046/j.1365-313X.2002.01400.x 12366800

[B71] Jing S. Zhou X. Song Y. Yu D. (2009). Heterologous expression of OsWRKY23 gene enhances pathogen defense and dark-induced leaf senescence in Arabidopsis. Plant Growth Regul. 58, 9. 10.1007/s10725-009-9366-z

[B72] Kamranfar I. Xue G. P. Tohge T. Sedaghatmehr M. Fernie A. R. Balazadeh S. (2018). Transcription factor RD26 is a key regulator of metabolic reprogramming during dark-induced senescence. New Phytol. 218, 1543–1557. 10.1111/nph.15127 29659022

[B73] Karpinska B. Zhang K. Rasool B. Pastok D. Morris J. Verrall S. R. (2018). The redox state of the apoplast influences the acclimation of photosynthesis and leaf metabolism to changing irradiance. Plant Cell Environ. 41, 1083–1097. 10.1111/pce.12960 28369975 PMC5947596

[B74] Kleffmann T. Russenberger D. Von Zychlinski A. Christopher W. Sjölander K. Gruissem W. (2004). The *Arabidopsis thaliana* chloroplast proteome reveals pathway abundance and novel protein functions. Curr. Biol. 14, 354–362. 10.1016/j.cub.2004.02.039 15028209

[B75] Kosová K. Vítámvás P. Prášil I. T. Renaut J. (2011). Plant proteome changes under abiotic stress—contribution of proteomics studies to understanding plant stress response. J. Proteomics 74, 1301–1322. 10.1016/j.jprot.2011.02.006 21329772

[B76] Kurkela S. Borg-Franck M. (1992). Structure and expression of kin2, one of two cold- and ABA-induced genes of Arabidopsis thaliana. Plant Mol. Biol. 19, 689–692. 10.1007/BF00026794 1627780

[B77] Lambertucci S. Orman K. M. Dasgupta S. Fisher J. P. Gazal S. Williamson R. J. (2019). Analysis of barley leaf epidermis and extrahaustorial proteomes during powdery mildew infection reveals that the PR5 thaumatin-like protein TLP5 is required for susceptibility towards Blumeria graminis f. sp. hordei. Front. In Plant Sci. 10, 1138. 10.3389/fpls.2019.01138 31736984 PMC6831746

[B78] Lara M. E. B. Garcia M.-C. G. Fatima T. Ehneß R. Lee T. K. Proels R. (2004). Extracellular invertase is an essential component of cytokinin-mediated delay of senescence. Plant Cell 16, 1276–1287. 10.1105/tpc.018929 15100396 PMC423215

[B79] Lebrasseur N. D. Macintosh G. C. Perez-Amador M. A. Saitoh M. Green P. J. (2002). Local and systemic wound-induction of RNase and nuclease activities in Arabidopsis: RNS1 as a marker for a JA-independent systemic signaling pathway. Plant J. 29, 393–403. 10.1046/j.1365-313x.2002.01223.x 11846873

[B80] Lee S. Lee E. J. Yang E. J. Lee J. E. Park A. R. Song W. H. (2004). Proteomic identification of annexins, calcium-dependent membrane binding proteins that mediate osmotic stress and abscisic acid signal transduction in Arabidopsis. Plant Cell 16, 1378–1391. 10.1105/tpc.021683 15161963 PMC490033

[B81] Levesque-Tremblay G. Pelloux J. Braybrook S. A. Müller K. (2015). Tuning of pectin methylesterification: consequences for cell wall biomechanics and development. Planta 242, 791–811. 10.1007/s00425-015-2358-5 26168980

[B82] Lippuner V. Chou I. T. Scott S. V. Ettinger W. F. Theg S. M. Gasser C. S. (1994). Cloning and characterization of chloroplast and cytosolic forms of cyclophilin from Arabidopsis thaliana. J. Biol. Chem. 269, 7863–7868. 8132503

[B83] Little D. Gouhier-Darimont C. Bruessow F. Reymond P. (2007). Oviposition by pierid butterflies triggers defense responses in Arabidopsis. Plant Physiol. 143, 784–800. 10.1104/pp.106.090837 17142483 PMC1803735

[B84] Liu W. X. Zhang F. C. Zhang W. Z. Song L. F. Wu W. H. Chen Y. F. (2013). Arabidopsis Di19 functions as a transcription factor and modulates PR1, PR2, and PR5 expression in response to drought stress. Mol. Plant 6, 1487–1502. 10.1093/mp/sst031 23404561

[B85] Lohaus G. Pennewiss K. Sattelmacher B. Hussmann M. Hermann Muehling K. (2001). Is the infiltration-centrifugation technique appropriate for the isolation of apoplastic fluid? A critical evaluation with different plant species. Physiol. Plant. 111, 457–465. 10.1034/j.1399-3054.2001.1110405.x 11299010

[B86] Mammarella N. D. Cheng Z. Fu Z. Q. Daudi A. Bolwell G. P. Dong X. (2015). Apoplastic peroxidases are required for salicylic acid-mediated defense against Pseudomonas syringae. Phytochemistry 112, 110–121. 10.1016/j.phytochem.2014.07.010 25096754 PMC4314520

[B87] Mano J. Nagata M. Okamura S. Shiraya T. Mitsui T. (2014). Identification of oxidatively modified proteins in salt-stressed Arabidopsis: a carbonyl-targeted proteomics approach. Plant Cell Physiol. 55, 1233–1244. 10.1093/pcp/pcu072 24850833

[B88] Martínez D. E. Guiamet J. J. (2014). Senescence-related changes in the leaf apoplast. J. Plant Growth Regul. 33, 44–55. 10.1007/s00344-013-9395-8

[B89] Martínez-González A. P. Ardila H. D. Martínez-Peralta S. T. Melgarejo-Muñoz L. Castillejo-Sánchez M. Jorrín-Novo J. V. (2018). What proteomic analysis of the apoplast tells us about plant–pathogen interactions. Plant Pathol. 67, 1647–1668. 10.1111/ppa.12893

[B90] Martinez D. E. Borniego M. L. Battchikova N. Aro E. M. Tyystjarvi E. Guiamet J. J. (2015). SASP, a Senescence-Associated Subtilisin Protease, is involved in reproductive development and determination of silique number in Arabidopsis. J. Exp. Bot. 66, 161–174. 10.1093/jxb/eru409 25371504

[B91] Masachis S. Segorbe D. Turrà D. Leon-Ruiz M. Fürst U. El Ghalid M. (2016). A fungal pathogen secretes plant alkalinizing peptides to increase infection. Nat. Microbiol. 1, 16043. 10.1038/nmicrobiol.2016.43 27572834

[B92] Mattsson M. Schjoerring J. K. (2003). Senescence-induced changes in apoplastic and bulk tissue ammonia concentrations of ryegrass leaves. New Phytol. 160, 489–499. 10.1046/j.1469-8137.2003.00902.x 33873655

[B93] Megel C. Hummel G. Lalande S. Ubrig E. Cognat V. Morelle G. (2019). Plant RNases T2, but not Dicer-like proteins, are major players of tRNA-derived fragments biogenesis. Nucleic Acids Res. 47, 941–952. 10.1093/nar/gky1156 30462257 PMC6344867

[B94] Mishina T. E. Zeier J. (2007). Pathogen-associated molecular pattern recognition rather than development of tissue necrosis contributes to bacterial induction of systemic acquired resistance in Arabidopsis. Plant J. 50, 500–513. 10.1111/j.1365-313X.2007.03067.x 17419843

[B95] Nardi C. F. Villarreal N. M. Rossi F. R. Martinez S. Martinez G. A. Civello P. M. (2015). Overexpression of the carbohydrate binding module of strawberry expansin2 in *Arabidopsis thaliana* modifies plant growth and cell wall metabolism. Plant Mol. Biol. 88, 101–117. 10.1007/s11103-015-0311-4 25837738

[B96] Nguyen-Kim H. San Clemente H. Balliau T. Zivy M. Dunand C. Albenne C. (2016). *Arabidopsis thaliana* root cell wall proteomics: Increasing the proteome coverage using a combinatorial peptide ligand library and description of unexpected Hyp in peroxidase amino acid sequences. Proteomics 16, 491–503. 10.1002/pmic.201500129 26572690

[B97] Noodén L. D. Guiamét J. J. John I. (1997). Senescence mechanisms. Physiol. Plant. 101, 746–753. 10.1111/j.1399-3054.1997.tb01059.x

[B98] Nouchi I. Hayashi K. Hiradate S. Ishikawa S. Fukuoka M. Chen C. P. (2012). Overcoming the difficulties in collecting apoplastic fluid from rice leaves by the infiltration-centrifugation method. Plant Cell Physiol. 53, 1659–1668. 10.1093/pcp/pcs102 22813544

[B99] O'brien J. A. Daudi A. Finch P. Butt V. S. Whitelegge J. P. Souda P. (2012). A peroxidase-dependent apoplastic oxidative burst in cultured Arabidopsis cells functions in MAMP-elicited defense. Plant Physiol. 158, 2013–2027. 10.1104/pp.111.190140 22319074 PMC3320203

[B100] O'leary B. M. Rico A. Mccraw S. Fones H. N. Preston G. M. (2014). The infiltration-centrifugation technique for extraction of apoplastic fluid from plant leaves using Phaseolus vulgaris as an example. J. Vis. Exp. e52113. 10.3791/52113 PMC4396939 25549068

[B101] O'leary B. M. Neale H. C. Geilfus C. M. Jackson R. W. Arnold D. L. Preston G. M. (2016). Early changes in apoplast composition associated with defence and disease in interactions between Phaseolus vulgaris and the halo blight pathogen Pseudomonas syringae pv. phaseolicola. Plant Cell Environ. 39, 2172–2184. 10.1111/pce.12770 27239727 PMC5026161

[B102] Oda-Yamamizo C. Mitsuda N. Sakamoto S. Ogawa D. Ohme-Takagi M. Ohmiya A. (2016). The NAC transcription factor ANAC046 is a positive regulator of chlorophyll degradation and senescence in Arabidopsis leaves. Sci. Rep. 6, 23609. 10.1038/srep35125 27021284 PMC4810360

[B103] Otegui M. S. (2018). Vacuolar degradation of chloroplast components: autophagy and beyond. J. Exp. Bot. 69, 741–750. 10.1093/jxb/erx234 28992297

[B104] Park S.-W. Li W. Viehhauser A. He B. Kim S. Nilsson A. K. (2013). Cyclophilin 20-3 relays a 12-oxo-phytodienoic acid signal during stress responsive regulation of cellular redox homeostasis. Proc. Natl. Acad. Sci. 110, 9559–9564. 10.1073/pnas.1218872110 23671085 PMC3677464

[B105] Petersen T. N. Brunak S. Von Heijne G. Nielsen H. (2011). SignalP 4.0: discriminating signal peptides from transmembrane regions. Nat. Methods 8, 785–786. 10.1038/nmeth1701 21959131

[B106] Piofczyk T. Jeena G. Pecinka A. (2015). Arabidopsis thaliana natural variation reveals connections between UV radiation stress and plant pathogen-like defense responses. Plant Physiol. Biochem. 93, 34–43. 10.1016/j.plaphy.2015.01.011 25656510

[B107] Potter S. Uknes S. Lawton K. Winter A. M. Chandler D. Dimaio J. (1993). Regulation of a hevein-like gene in Arabidopsis. Mol. Plant-Microbe Interact. 6, 680–685. 10.1094/MPMI-6-680 8118053

[B108] Provart N. J. Gil P. Chen W. Han B. Chang H. S. Wang X. (2003). Gene expression phenotypes of Arabidopsis associated with sensitivity to low temperatures. Plant Physiol. 132, 893–906. 10.1104/pp.103.021261 12805619 PMC167029

[B109] Rascio A. Platani C. Di Fonzo N. Wittmer G. (1992). Bound water in durum wheat under drought stress. Plant Physiol. 98, 908–912. 10.1104/pp.98.3.908 16668763 PMC1080286

[B110] Rizhsky L. Liang H. Shuman J. Shulaev V. Davletova S. Mittler R. (2004). When defense pathways collide. The response of Arabidopsis to a combination of drought and heat stress. Plant Physiol. 134, 1683–1696. 10.1104/pp.103.033431 15047901 PMC419842

[B111] Robinson W. D. Carson I. Ying S. Ellis K. Plaxton W. C. (2012). Eliminating the purple acid phosphatase AtPAP26 in Arabidopsis thaliana delays leaf senescence and impairs phosphorus remobilization. New Phytol. 196, 1024–1029. 10.1111/nph.12006 23072540

[B112] Rodríguez-Celma J. Ceballos-Laita L. Grusak M. A. Abadía J. López-Millán A.-F. (2016). Plant fluid proteomics: delving into the xylem sap, phloem sap and apoplastic fluid proteomes. Biochim. Biophys. Acta 1864, 991–1002. 10.1016/j.bbapap.2016.03.014 27033031

[B113] Rutter B. D. Innes R. W. (2017). Extracellular vesicles isolated from the leaf apoplast carry stress-response proteins. Plant Physiol. 173, 728–741. 10.1104/pp.16.01253 27837092 PMC5210723

[B114] Sävenstrand H. Brosché M. Strid A. (2004). Ultraviolet-B signalling: Arabidopsis brassinosteroid mutants are defective in UV-B regulated defence gene expression. Plant Physiol. Biochem. 42, 687–694. 10.1016/j.plaphy.2004.06.011 15474373

[B115] Sattelmacher B. Horst W. J. (2007). The apoplast of higher plants: compartment of storage, transport and reactions: the significance of the apoplast for the mineral nutrition of higher plants (Dordrecht, The Netherlands: Springer Science & Business Media). 10.1007/978-1-4020-5843-1

[B116] Schippers J. H. Schmidt R. Wagstaff C. Jing H. C. (2015). Living to die and dying to live: the survival strategy behind leaf senescence. Plant Physiol. 169, 914–930. 10.1104/pp.15.00498 26276844 PMC4587445

[B117] Seo P. J. Lee A.-K. Xiang F. Park C.-M. (2008). Molecular and functional profiling of Arabidopsis pathogenesis-related genes: insights into their roles in salt response of seed germination. Plant Cell Physiol. 49, 334–344. 10.1093/pcp/pcn011 18203731

[B118] Soares J. R. Jose Tenorio De Melo E. Da Cunha M. Fernandes K. V. S. Taveira G. B. Da Silva Pereira L. (2017). Interaction between the plant ApDef1 defensin and Saccharomyces cerevisiae results in yeast death through a cell cycle- and caspase-dependent process occurring *via* uncontrolled oxidative stress. Biochim. Biophys. Acta 1861, 3429–3443. 10.1016/j.bbagen.2016.09.005 27614033

[B119] Song W. Mentink R. A. Henquet M. G. Cordewener J. H. Van Dijk A. D. Bosch D. (2013). N-glycan occupancy of Arabidopsis N-glycoproteins. J. Proteomics 93, 343–355. 10.1016/j.jprot.2013.07.032 23994444

[B120] Suzuki N. Rizhsky L. Liang H. Shuman J. Shulaev V. Mittler R. (2005). Enhanced tolerance to environmental stress in transgenic plants expressing the transcriptional coactivator multiprotein bridging factor 1c. Plant Physiol. 139, 1313–1322. 10.1104/pp.105.070110 16244138 PMC1283768

[B121] Taira M. Valtersson U. Burkhardt B. Ludwig R. A. (2004). Arabidopsis thaliana GLN2-encoded glutamine synthetase is dual targeted to leaf mitochondria and chloroplasts. Plant Cell 16, 2048–2058. 10.1105/tpc.104.022046 15273293 PMC519197

[B122] Tetlow I. Farrar J. (1993). Apoplastic sugar concentration and pH in barley leaves infected with brown rust. J. Exp. Bot. 44, 929–936. 10.1093/jxb/44.5.929

[B123] Tran H. T. Plaxton W. C. (2008). Proteomic analysis of alterations in the secretome of Arabidopsis thaliana suspension cells subjected to nutritional phosphate deficiency. Proteomics 8, 4317–4326. 10.1002/pmic.200800292 18814331

[B124] Tran H. T. Qian W. Hurley B. A. She Y. M. Wang D. Plaxton W. C. (2010). Biochemical and molecular characterization of AtPAP12 and AtPAP26: the predominant purple acid phosphatase isozymes secreted by phosphate-starved *Arabidopsis thaliana* . Plant Cell Environ. 33, 1789–1803. 10.1111/j.1365-3040.2010.02184.x 20545876

[B125] Trusova S. V. Chichkova N. V. Vartapetian A. B. (2019). Sometimes they come back: endocytosis provides localization dynamics of a subtilase in cells committed to cell death. J. Exp. Bot. 70, 2003–2007. 10.1093/jxb/erz014 30668760 PMC6460962

[B126] Tyree M. (1976). Negative turgor pressure in plant cells: fact or fallacy? Can. J. Bot. 54, 2738–2746. 10.1139/b76-294

[B127] Uknes S. Mauch-Mani B. Moyer M. Potter S. Williams S. Dincher S. (1992). Acquired resistance in Arabidopsis. Plant Cell 4, 645–656. 10.1105/tpc.4.6.645 1392589 PMC160161

[B128] Van Der Graaff E. Schwacke R. Schneider A. Desimone M. Flügge U.-I. Kunze R. (2006). Transcription analysis of Arabidopsis membrane transporters and hormone pathways during developmental and induced leaf senescence. Plant Physiol. 141, 776–792. 10.1104/pp.106.079293 16603661 PMC1475451

[B129] Van Loon L. Van Strien E. (1999). The families of pathogenesis-related proteins, their activities, and comparative analysis of PR-1 type proteins. Physiol. Mol. Plant Pathol. 55, 85–97. 10.1006/pmpp.19990213

[B130] Velazhahan R. Datta S. K. Muthukrishnan S. (1999). “The PR-5 family: thaumatin-like proteins,” in Pathogenesis-related proteins in plants. Eds. Datta S. K. Muthukrishnan S. (Boca Raton: CRC Press), 107–129.

[B131] Wardlaw I. F. (2005). Consideration of apoplastic water in plant organs: a reminder. Funct. Plant Biol. 32, 561–569. 10.1071/FP04127 32689156

[B132] Wei C. Lintilhac P. M. (2007). Loss of stability: a new look at the physics of cell wall behavior during plant cell growth. Plant Physiol. 145, 763–772. 10.1104/pp.107.101964 17905864 PMC2048773

[B133] Wei H. Brunecky R. Donohoe B. S. Ding S.-Y. Ciesielski P. N. Yang S. (2015). Identifying the ionically bound cell wall and intracellular glycoside hydrolases in late growth stage Arabidopsis stems: implications for the genetic engineering of bioenergy crops. Front. In Plant Sci. 6, 315. 10.3389/fpls.2015.00315 26029221 PMC4429552

[B134] Winter D. Vinegar B. Nahal H. Ammar R. Wilson G. V. Provart N. J. (2007). An “Electronic Fluorescent Pictograph” browser for exploring and analyzing large-scale biological data sets. PloS One 2, e718. 10.1371/journal.pone.0000718 17684564 PMC1934936

[B135] Wu Y. Yang T. Song Y. Zhang X. Xu S. Xue G. (2016). Metabolic regulation of ammonia emission in different senescence phenotypes of Nicotiana tabacum. Biol. Plant. 60, 190–194. 10.1007/s10535-015-0556-4

[B136] Zörb C. Mühling K. H. Kutschera U. Geilfus C.-M. (2015). Salinity stiffens the epidermal cell walls of salt-stressed maize leaves: is the epidermis growth-restricting? PloS One 10, e0118406. 10.1371/journal.pone.0118406 25760715 PMC4356557

[B137] Zabaleta E. Oropeza A. Assad N. Mandel A. Salerno G. Herrera-Estrella L. (1994). Antisense expression of chaperonin 60β in transgenic tobacco plants leads to abnormal phenotypes and altered distribution of photoassimilates. Plant J. 6, 425–432. 10.1046/j.1365-313X.1994.06030425.x

[B138] Zavaliev R. Levy A. Gera A. Epel B. L. (2013). Subcellular dynamics and role of Arabidopsis beta-1,3-glucanases in cell-to-cell movement of tobamoviruses. Mol. Plant Microbe Interact. 26, 1016–1030. 10.1094/MPMI-03-13-0062-R 23656331

[B139] Zhang L. Cheng J. Sun X. Zhao T. Li M. Wang Q. (2018). Overexpression of VaWRKY14 increases drought tolerance in Arabidopsis by modulating the expression of stress-related genes. Plant Cell Rep. 37, 1159–1172. 10.1007/s00299-018-2302-9 29796948

[B140] Zhao J. Wang J. An L. Doerge R. W. Chen Z. J. Grau C. R. (2007). Analysis of gene expression profiles in response to Sclerotinia sclerotiorum in Brassica napus. Planta 227, 13–24. 10.1007/s00425-007-0586-z 17665211

[B141] Zhao Y. Chan Z. Gao J. Xing L. Cao M. Yu C. (2016). ABA receptor PYL9 promotes drought resistance and leaf senescence. Proc. Natl. Acad. Sci. 113, 1949–1954. 10.1073/pnas.1522840113 26831097 PMC4763734

[B142] Zhao P. Zhang F. Liu D. Imani J. Langen G. Kogel K. H. (2017). Matrix metalloproteinases operate redundantly in Arabidopsis immunity against necrotrophic and biotrophic fungal pathogens. PloS One 12, e0183577. 10.1371/journal.pone.0183577 28832648 PMC5568438

[B143] Zhou Q. Yu Q. Wang Z. Pan Y. Lv W. Zhu L. (2013). Knockdown of GDCH gene reveals reactive oxygen species-induced leaf senescence in rice. Plant Cell Environ. 36, 1476–1489. 10.1111/pce.12078 23421602

[B144] Ziemann S. Linde K. Lahrmann U. Acar B. Kaschani F. Colby T. (2018). An apoplastic peptide activates salicylic acid signalling in maize. Nat. Plants 4, 172–180. 10.1038/s41477-018-0116-y 29483684

