# Supplementary material for: Physiological and Proteomic Changes in the Apoplast Accompany Leaf Senescence in Arabidopsis
Source: Front Plant Sci. 2020 Jan 8;10:1635. doi: 10.3389/fpls.2019.01635 (PMC6960232; doi:10.3389/fpls.2019.01635)
Supplement: Supplementary file 1 [file DataSheet_1.doc]

Supplementary Table S1

| **Supplementary Table S1.**  **AF proteins identified in the 37 kDa bands (Figure 5B).** | | | | | | | |
| --- | --- | --- | --- | --- | --- | --- | --- |
| Accession | Protein name | Coverage S2 | # PSM  S2 | Coverage S3 | # PSM  S3 | N-g.s. | MW [kDa] |
| **P33157** | **Glucan endo-1,3-beta-glucosidase (PR2)** | **15.63** | **6** | **46.90** | **45** | **YES** | **37.3** |
| O24603 | Chitinase class 4-like protein (LSC222) |  |  | 17.33 | 3 | YES | 29.8 |
| Q93V74 | CYCLASE1 | 13.33 | 3 |  |  | YES | 28.4 |
| P28493 | Pathogenesis-related protein 5 (PR5) | 5.86 | 1 | 12.55 | 2 | YES | 25.2 |
| Q9LJR2 | Lectin-like protein (LEC) |  |  | 9.23 | 3 | YES | 29.7 |
| Q43387 | Peroxidase 71 (PER71) |  |  | 9.15 | 3 | YES | 34.9 |
| O65469 | Putative cysteine-rich receptor-like protein kinase 9 (EP1) |  |  | 4.53 | 1 | YES | 29.7 |
| Q9M2U5 | Endochitinase (EP3) |  |  | 4.40 | 1 | YES | 29.4 |
| Q94BT2 | Auxin-induced in root cultures protein 12 (AIR12) |  |  | 3.97 | 1 | YES | 25.6 |
| F4HR96 | Non-lysosomal glucosylceramidase |  |  | 3.38 | 2 | YES | 105.7 |
| Q9FFH6 | Fasciclin-like arabinogalactan protein 13 (FLA13) |  |  | 3.24 | 1 | YES | 26.2 |
| F4IGL7 | Fructose-bisphosphate aldolase (FBA1) |  |  | 3.22 | 1 | YES | 33.3 |
| O24598 | Endochitinase At2g43580 |  |  | 3.02 | 1 | YES | 28.8 |
| F4J8V9 | Actin 2 (ACT2) |  |  | 2.70 | 1 | YES | 41.2 |
| Q9C7M2 | Slow embryo development 1 (SED1) | 1.30 | 3 | 1.30 | 1 | YES | 94.8 |
| Mass spectrometry analysis was performed on the 37 kDa protein bands shown in figure 5B. Proteins identified with at least one unique peptide are shown. The most abundant protein is shown in bold. Proteins with potential N-glycosylation sites (N-g.s.) are indicated in the column "N-g.s." | | | | | | | |

Supplementary Table S2

| **Supplementary Table S2. AF proteins identified in the 25 kDa bands (Figure 5B)** | | | | | | | |
| --- | --- | --- | --- | --- | --- | --- | --- |
| Accession | Protein name | Coverage S2 | # PSM  S2 | Coverage S3 | # PSM  S3 | N-g.s. | MW [kDa] |
| **P28493** | **Pathogenesis-related protein 5 (PR5)** | **58.58** | **27** | **58.58** | **50** | **YES** | **25.2** |
| P33157 | Glucan endo-1,3-beta-glucosidase, acidic isoform (PR2) |  |  | 14.75 | 3 | YES | 37.3 |
| Q94BT2 | Auxin-induced in root cultures protein 12 (AIR12) | 3.97 | 1 | 3.97 | 1 | YES | 25.6 |
| P59833 | Uncharacterized GPI-anchored protein At5g19250 |  |  | 3.57 | 1 | YES | 21.0 |
| Q9C7M2 | Slow embryo development 1 (SED1) |  |  | 1.30 | 1 | YES | 94.8 |
| Mass spectrometry analysis was performed on the 25 kDa protein bands shown in figure 5B. Proteins identified with at least one unique peptide are shown. The most abundant protein is shown in bold. Proteins with potential N-glycosylation sites (N-g.s.) are indicated in the column "N-g.s." | | | | | | | |

**Supplementary Table S3. AF proteins whose levels decrease significantly more than twofold during leaf senescence.**

| **Protein** | | **Protein ID** | **Gen ID** | **MW (kDa)** | **SL** | **Biological process** | **Biotic/ abiotic stress associated** | **gene expression during senescence** | **SP** | **N-g.s.** | **CL** |
| --- | --- | --- | --- | --- | --- | --- | --- | --- | --- | --- | --- |
| Carbonic anhydrase (CA2) | | A8MQY4 | At5g14740 | 34.4 | **Ap, EV, Mit, G, T, V, MP, Chl**, **Cyt,** N | Defense response to bacteria | B | ↓ | NO | YES | ** |
| Aldolase-type TIM barrel family protein (GOX1) | | A8MS37 | At3g14420 | 39.4 | **Ap, Mit, MP, Chl, PX, N, M,** Cyt | Defense response to bacteria, photorespiration, oxidation-reduction process, hydrogen peroxide biosynthesis | B | ↓ | NO | YES | ** |
| Bifunctional inhibitor/lipid-transfer protein/seed storage 2S albumin superfamily protein (LTPG6) | | F4I083 | At1g55260 | 25.0 | **EV, N, MP, M,** Cyt, Mit | unknown |  | ↓ | NO | YES | ** |
| Lactoylglutathione lyase GLX1 (GLX1) | | O65398 | At1g11840 | 31.9 | **Ap**, **Cyt**, **Mit**, **PX**, **PM**, **Chl**, **V,** N | Carbohydrate metabolism, oxidation-reduction process |  | ↓ | NO | YES | *** |
| Beta-galactosidase 8 (BGAL8) | | F4IIQ3 | At2g28470 | 92.5 | **Ap**, **V**, ER, G, Mit, N, Chl, | Carbohydrate metabolism |  | ↓ | YES | YES | ** |
| Sinapoylglucose 1 (SNG1) | | F4IKK4 | At2g22990 | 36.7 | **Ap, V**, Cyt, ER, G, PM | Phenylpropanoid metabolism, proteolysis |  | ↓ | NO | YES | ** |
| Peptidyl-prolyl cis-trans isomerase (CYP20-3, ROC4) | | F4IX28 | At3g62030 | 28.1 | **Ap, Mit, PM, Cyt, Chl,** ER, N | Cysteine biosynthesis, defense response to bacteria, protein peptidyl-prolyl isomerization, protein refolding, response to light intensity, response to oxidative stress, response to salt stress, signal transduction | B - A | ↓ | NO | YES | ** |
| Probable glucan endo-1,3-beta-glucosidase BG3 (BG3) | | F4J270 | At3g57240 | 37.6 | **AP**, **PM, V**, **ER**, G, Mit, Chl | Defense response to bacteria, carbohydrate metabolism | B | ↓ | YES | YES | *** |
| Transketolase (TKL1) | | F4JBY2 | At3g60750 | 79.8 | **Ap**, **EV**, **T**, **G**, **Mit**, **PM**, Chl, V, Cyt | Pentose phosphate cycle, response to salt stress | A | ↓ | NO | YES | ** |
| Receptor Serine/Threonine kinase-like protein | | F4JQT1 | At4g18250 | 95.2 | **PM, M,** Ap, Cyt, ER, G, N, Chl | Protein phosphorylation |  | ↑ | NO | YES | *** |
| Early nodulin-like protein 19 (ENODL19) | | F4JRH9 | At4g12880 | 12.0 | **Ap**, **G**, **PM**, Mit, Chl, ER | Electron transport chain |  | ↓ | NO | YES | ** |
| Aspartate aminotransferase (ASP5) | | F4JTH0 | At4g31990 | 49.3 | **Ap, Mit, Chl, amyloplast** | Amino acid metabolism, response to cold | A | ↓ | NO | YES | ** |
| Ferredoxin--NADP reductase (FNR1) | | F4JZ46 | At5g66190 | 29.7 | **Ap**, **T**, **N, Mit, G**, **Chl**, **PM**, Cyt | Oxidation-reduction process, defense response to bacteria, electron transport chain | B | ↓ | NO | YES | ** |
| Ribulose bisphosphate carboxylase large chain (RBCL) | | O03042 | AtCg00490 | 52.9 | **Ap**, **EV,** **G**, **Mit**, **N**, **PM**, **M**, **Chl**, **T,** Cyt | Carbon fixation, oxidation-reduction process, photorespiration, pentose-phosphate cycle |  | ↓ | NO | YES | **** |
| Aspartyl protease AED3 (AED3) | | O04496 | At1g09750 | 47.6 | **Ap**, **G**, **Mit**, **T**, **PM**, Chl, N, V, ER | Proteolysis, systemic acquired resistance, regulation of programmed cell death | B | ↓ | YES | YES | **** |
| Calvin cycle protein CP12-1 (CP12-1) | | O22914 | At2g47400 | 13.5 | **Chl**, Ap, ER, Cyt, G, Mit, N | Negative regulation of reductive pentose-phosphate cycle |  | ↓ | NO | NO | ** |
| Serine hydroxymethyltransferase 4 (SHM4) | | O23254 | At4g13930 | 51.7 | **Ap**, **Cyt**, **G**, **Mit**, **PM**, **M**, **Chl,** N | Circadian rhythm, folic acid metabolic process, amino acid metabolism |  | ↓ | NO | YES | ** |
| Thioredoxin M1 (THM1) | | O48737 | At1g03680 | 19.7 | **Ap**, **Mit, Chl** | Cell redox homeostasis, oxidation-reduction process, regulation of carbohydrate metabolism, response to cold, response to oxidative stress, glycerol ether metabolism | A | ↓ | NO | YES | **** |
| Plant invertase/pectin methylesterase inhibitor superfamily protein | | O49297 | At1g23205 | 22.7 | **Ap**, ER, G, N, PM, Chl, v | Negative regulation of catalytic activity |  | ↓ | YES | YES | ** |
| 20 kDa chaperonin (CPN20) | | O65282 | At5g20720 | 26.8 | **Ap, G, Mit**, **PX**, **Chl, Cyt** | Protein refolding, negative regulation of abscisic acid-activated signaling pathway, positive regulation of superoxide dismutase activity, protein heterotetramerization, response to cold |  | ↓ | NO | NO | ** |
| Subtilisin-like protease SBT1.7 (SBT1.7) | | O65351 | At5g67360 | 79.4 | **Ap, G, T**, ER, Mit, PM, Chl, V | Mucilage metabolic process involved in seed coat development, proteolysis |  | ─ | YES | YES | *** |
| Putative cysteine-rich receptor-like protein kinase 9 (CRK9, EP1) | | O65469 | At4g23170 | 29.7 | **Ap**, PM, G, Chl, Mit, ER | Programmed cell death, systemic acquired resistance | B | no inf. | YES | YES | ** |
| Superoxide dismutase [Cu-Zn] 2 (CSD2) | | O78310 | At2g28190 | 22.2 | **Ap**, **Chl,** Mit | Response to UV-B, response to light intensity, response to oxidative stress, response to ozone, response to salt stress, gene silencing by miRNA, oxidation-reduction process, removal of superoxide radicals | A | ↓ | NO | YES | *** |
| Pectin acetylesterase 3 (PAE3) | | O80731 | At2g46930 | 45.9 | **Ap**, **PM**, ER, G, Mit, N, V | Cell wall organization |  | ↓ | YES | YES | ** |
| NAD(P)-binding Rossmann-fold superfamily protein | | O80934 | At2g37660 | 34.9 | **Ap, Mit, Chl** | Defense response to bacteria | B | ↓ | NO | NO | ** |
| Pectin lyase-like superfamily protein | | O81746 | At4g23500 | 54.7 | **Ap**, Mit, Chl, G, ER, PM, Cyt | Carbohydrate metabolism |  | no inf. | YES | YES | ** |
| Phosphoglycolate phosphatase 1A (PGLP1A) | | P0DKC3 | At5g36700 | 39.7 | **Chl**, Mit, N, PM, V, Cyt | Dephosphorylation, photorespiration |  | ↓ | NO | YES | ** |
| Ribulose bisphosphate carboxylase small chain 1A (RBCS-1A) | | P10795 | At1g67090 | 20.2 | **Ap**, **EV, G,** **Mit, N**, **PX**, **PM**, **Chl** | Chloroplast ribulose bisphosphate carboxylase complex assembly, oxidation-reduction process, photorespiration, photosynthesis, reductive pentose-phosphate cycle, response to light, response to cold |  | ↓ | NO | NO | **** |
| Ribulose bisphosphate carboxylase small chain 1B (RBCS-1B) | | P10796 | At5g38430 | 20.3 | **Ap**, **PM, M, Chl**, ER, G, Mit, N, | Oxidation-reduction process, photorespiration, photosynthesis, reductive pentose-phosphate cycle, response to light |  | ↓ | NO | NO | **** |
| Ribulose bisphosphate carboxylase small chain 2B (RBCS-2B) | | P10797 | At5g38420 | 20.3 | **Ap**, **PM, M, Chl, G**, Mit, **N** | Oxidation-reduction process, photorespiration, photosynthesis, reductive pentose-phosphate cycle, response to light |  | ↓ | NO | NO | *** |
| Ribulose bisphosphate carboxylase/oxygenase activase (RCA) | | P10896 | At2g39730 | 51.9 | **Ap, G, Chl, Mit**, **T**, **N**, **PM**, **M**, Cyt | Defense response to bacteria, leaf senescence, response to cold, response to light | B - A | ↓ | NO | YES | **** |
| Chaperonin 60 subunit alpha 1 (CPN60A1) | | P21238 | At2g28000 | 62.0 | **Ap, G**, **Mit**, **T**, **N**, **PM, M, Chl**, **V,** Cyt | Chloroplast organization, embryo development, protein folding, protein import into mitochondrial intermembrane space, protein refolding |  | ↓ | NO | YES | *** |
| Chaperonin 60 subunit beta 1 (CPN-60 β1) | | P21240 | At1g55490 | 63.8 | **Ap**, **T**, **G**, **M**, **N**, **PM**, **Chl,** Mit | Cell death, protein folding, protein import into mitochondrial intermembrane space, protein refolding, response to cold, systemic acquired resistance | B - A | ↓ | NO | YES | ** |
| Oxygen-evolving enhancer protein 1-1 (OEE1) | | P23321 | At5g66570 | 35.1 | **Ap**, **Mit, G, T,** **PM**, **M,** **Chl** | Defense response to bacteria, photosynthesis, photosystem II assembly and stabilization, regulation of protein dephosphorylation | B | ↓ | NO | NO | *** |
| Phosphoribulokinase (PRK) | | P25697 | At1g32060 | 44.4 | **Ap**, **Mit**, **Cyt**, **N**, **PM**, **M, Chl** | Photosynthesis, defense response to bacteria, phosphorylation, reductive pentose-phosphate cycle, response to cold | B - A | ↓ | NO | YES | ** |
| Fructose-1,6-bisphosphatase 1 (FBPase1) | | P25851 | At3g54050 | 45.1 | **Ap, Mit, PM**, **Chl, Cyt,** N | Gluconeogenesis, photosynthesis, photosynthetic electron transport in photosystem I, reductive pentose-phosphate cycle, response to cold, sucrose biosynthesis |  | ↓ | NO | YES | *** |
| Glycine cleavage system H protein 1 (GDH1, GDCH) | | P25855 | At2g35370 | 17.9 | **Mit, N, Chl** | Glycine decarboxylation, photorespiration |  | ↓ | NO | YES | ** |
| Glyceraldehyde-3-phosphate dehydrogenase (GAPB) | | P25857 | At1g42970 | 47.6 | **Ap, G**, **Mit, T, N**, **PM**, **M**, **Chl,** Cyt | Glucose metabolism, oxidation-reduction process, reductive pentose-phosphate cycle, response to cold, response to light, response to sucrose | A | ↓ | NO | YES | ** |
| Beta carbonic anhydrase 1 (BCA1) | | P27140 | At3g01500 | 37.4 | **G, Mit, N, PM, M, Chl, Ap,** Cyt, ER | Carbon utilization, defense response to bacteria, defense response to fungus, negative regulation of stomatal complex development, photosynthesis, regulation of stomatal movement, response to cold | B | ↓ | NO | YES | *** |
| Stress-induced protein KIN2 | | P31169 | At5g15970 | 6.5 | **PX, PM, Chl, Cyt, N,** Mit, Ap | Response to cold, response to osmotic stress, response to water deprivation | A | ─ | NO | NO | **** |
| Peptidyl-prolyl cis-trans isomerase (CYP18-3) | | P34790 | At4g38740 | 18.4 | **Ap, Cyt, Mit, PX, PM, Chl,** N, G, ER | Blue light signaling pathway, brassinosteroid mediated signaling pathway, de-etiolation, hypersensitive response, protein peptidyl-prolyl isomerization, protein refolding, red, far-red light phototransduction, regulation of protein phosphorylation, response to light, signal transduction | B | ↓ | NO | YES | ** |
| Myrosinase 1 (TGG1) | | P37702 | At5g26000 | 61.1 | **Ap, ER, V, T, PX, Chl, PM, N, Mit**, Cyt, G | Abscisic acid-activated signaling pathway, carbohydrate metabolism, defense response to insect, glucosinolate catabolism, regulation of stomatal movement, response to salt stress, response to abscisic acid | B - A | ↓ | YES | YES | **** |
| Probable cinnamyl alcohol dehydrogenase 9 (CAD9) | | P42734 | At4g39330 | 38.9 | **Ap, PM, Cyt, T,** Mit, N, Chl | Lignin biosynthesis, oxidation-reduction process |  | ↓ | NO | YES | ** |
| Beta carbonic anhydrase 2 (BCA2) | | P42737 | At5g14740 | 36.6 | **Ap, EV, N, G, PM, Chl, T, Cyt, Mit,** ER | Carbon utilization, defense response to bacteria | B | ↓ | NO | YES | ** |
| Glutamate-1-semialdehyde 2,1-aminomutase 1 (GSA 1) | | P42799 | At5g63570 | 50.3 | **Ap, Mit, Chl,** PX, Cyt | Chlorophyll biosynthesis, porphyrin-containing compound biosynthesis, protoporphyrinogen IX biosynthesis, response to light |  | ↓ | NO | NO | ** |
| Sedoheptulose-1,7-bisphosphatase (SBPase) | | P46283 | At3g55800 | 42.4 | **Ap, G, N, PM, Chl,** Mit | Carbohydrate biosynthesis, defense response to bacteria, gluconeogenesis, reductive pentose-phosphate cycle, starch biosynthesis, sucrose biosynthesis |  | ↓ | NO | YES | *** |
| Cysteine synthase 1 (OASA1, OLD3) | | P47998 | At4g14880 | 33.8 | **Ap, EV, G, Cyt, Mit, N, PX, PM, M, Chl, V**, **T**, ER | Aging, cysteine biosynthesis, double fertilization, pollen tube growth |  | ↓ | NO | NO | ** |
| Cysteine synthase, chloroplastic/chromoplastic (OASB) | | P47999 | At2g43750 | 41.6 | **Ap, G, Mit, PM, Chl**, Cyt | Cysteine biosynthesis, double fertilization, pollen tube growth |  | ↓ | NO | NO | ** |
| Triosephosphate isomerase (TIM) | | P48491 | At3g55440 | 27.2 | **Ap, EV, Cyt, G, Mit, PM, Chl, V**, **T,** PX, N | Carbohydrate metabolism, gluconeogenesis, glyceraldehyde-3-phosphate biosynthesis, glycerol catabolism, glycolysis, response to salt stress | A | ↓ | NO | YES | **** |
| Glucose-1-phosphate adenylyltransferase small subunit (ADG1) | | P55228 | At5g48300 | 56.6 | **Ap, G, Mit, Chl**, Cyt | Glycogen biosynthesis, photoperiodism, flowering, starch biosynthetic process |  | ↓ | NO | YES | ** |
| Xyloglucan endotransglucosylase/hydrolase protein 22 (XTH-22) | | Q38857 | At5g57560 | 32.1 | **Ap, G, Chl**, Mit, Cyt, ER, N | Cell wall biogenesis, cell wall organization, response to cold, response to heat, response to mechanical stimulus, xyloglucan metabolism |  | ↓ | YES | YES | ** |
| Peptidyl-prolyl cis-trans isomerase (CYP19-1, ROC3) | | Q38900 | At2g16600 | 18.5 | **Ap, EV, Cyt, G, Mit, PM, Chl, PX**, N | Protein peptidyl-prolyl isomerization, protein refolding, signal transduction |  | ↓ | NO | YES | *** |
| Xyloglucan endotransglucosylase/hydrolase protein 4 (XTH-4) | | Q39099 | At2g06850 | 34.3 | **Ap, Cyt, G, Mit, PM, M, Chl, T,** V, ER | Cell wall biogenesis, cell wall organization, response to low light intensity stimulus, response to mechanical stimulus, unidimensional cell growth, xyloglucan metabolic process | A | ↓ | YES | YES | ** |
| Ferredoxin--nitrite reductase (NIR1) | | Q39161 | At2g15620 | 65.5 | **Ap, G, Mit, PM, M, Chl**, Cyt | Nitrate assimilation, oxidation-reduction process, response to nitrate | A | ↓ | NO | NO | ** |
| Oxygen-evolving enhancer protein 2-1 (OEE2) | | Q42029 | At1g06680 | 28.1 | **Ap, G, Mit, Chl, M,** Cyt, N | Defense response to bacteria, photosynthesis | B | ↓ | NO | NO | **** |
| Peptidyl-prolyl cis-trans isomerase (CYP18-4) | | Q42406 | At4g34870 | 18.4 | **Ap, Cyt, G, Mit, PX, PM, Chl, V, T, N** | Protein peptidyl-prolyl isomerization, protein refolding, signal transduction |  | ↓ | NO | YES | ** |
| Profilin-2 (PRO2) | | Q42418 | At4g29350 | 14.0 | **Ap, PM, Chl, Cyt, ER, N**, Mit, G | Actin polymerization or depolymerization, inflorescence development, lateral root development, leaf development, sequestering of actin monomers |  | ↑ | NO | NO | ** |
| Glutamate-1-semialdehyde 2,1-aminomutase 2 (GSA 2) | | Q42522 | At3g48730 | 50.1 | **G, Chl, T, Cyt**, PX, Mit | Chlorophyll biosynthesis, porphyrin-containing compound biosynthesis |  | ↓ | NO | NO | ** |
| Non-specific lipid-transfer protein 1 (LTP 1) | | Q42589 | At2g38540 | 11.7 | **Ap, G, PX, PM, Chl, T, Cyt**, Mit, ER, V | Cell wall organization, lipid transport |  | ↓ | YES | NO | *** |
| Glutamine synthetase (GS2) | | Q43127 | At5g35630 | 47.4 | **Ap, G, Mit, PM, Chl, T**, Cyt | Aging, ammonia assimilation cycle, glutamine biosynthesis |  | ↓ | NO | YES | *** |
| Porphobilinogen deaminase (PBG) | | Q43316 | At5g08280 | 41.0 | **Ap, G, Chl, T**, Mit, Cyt | Chlorophyll biosynthesis, chloroplast RNA modification, defense response to bacteria, heme biosynthesis, peptidyl-pyrromethane cofactor linkage, porphyrin-containing compound biosynthesis |  | ↓ | NO | NO | ** |
| Serine--glyoxylate aminotransferase (AGT) | | Q56YA5 | At2g13360 | 44.2 | **Ap, Mit, PX, PM, M, Chl**, Cyt, N | Glycine biosynthesis, photorespiration |  | ↓ | NO | YES | ** |
| Glycerophosphodiester phosphodiesterase (GDPDL1) | | Q7Y208 | At1g66970 | 83.7 | **Ap, EV, PM, M, Chl**, G, ER | Cellular phosphate ion homeostasis, glycerol metabolism, lipid metabolism |  | ↓ | YES | YES | ** |
| Cysteine proteinase inhibitor 4 (CYS4) | | Q84WT8 | At4g16500 | 12.5 | **Ap, Cyt, V, T, Chl**, G, ER, PM, Mit | Defense response, negative regulation of endopeptidase activity | B | ↓ | YES | YES | ** |
| Eukaryotic aspartyl protease family protein | | Q84WU7 | At3g51330 | 57.8 | **Ap, EV, PM**, **M**, ER, Mit, G, V, Cyt | Proteolysis |  | ↑ | YES | YES | ** |
| Alpha-L-fucosidase 1 (AtFUC1) | | Q8GW72 | At2g28100 | 57.2 | **Ap, V**, PM, Mit, Chl, ER, G, Cyt | [Carbohydrate metabolism](https://www.arabidopsis.org/servlets/TairObject?type=keyword&id=5811), glycoprotein catabolism, [glycoside catabolism](https://www.arabidopsis.org/servlets/TairObject?type=keyword&id=5927) |  | ↓ | YES | YES | **** |
| Serine carboxypeptidase-like 20 (SCPL20) | | Q8L7B2 | At4g12910 | 55.8 | **Ap, PX, V, Cyt,** ER, G, PM | Proteolysis, secondary metabolism |  | ↑ | YES | YES | *** |
| Probable prolyl 4-hydroxylase (AtP4H4) | | Q8LAN3 | At5g18900 | 33.0 | **ER, G**, **Cyt, N, Ap**, PM, M, Mit, Chl | Oxidation-reduction process, peptidyl-proline hydroxylation to 4-hydroxy-L-proline |  | ↑ | YES | YES | ** |
| S-formylglutathione hydrolase (AtSFGH) | | Q8LAS8 | At2g41530 | 31.6 | **Ap, Mit, PM**, Cyt, N, Chl | Formaldehyde catabolism |  | ↓ | NO | YES | ** |
| Peptidyl-prolyl cis-trans isomerase (CYP23) | | Q8LDR3 | At1g26940 | 25.5 | **G**, Ap, Mit, Cyt, V, ER | protein peptidyl-prolyl isomerization, |  | ─ | YES | NO | ** |
| Probable xyloglucan endotransglucosylase/hydrolase protein 6 (XTH-6) | | Q8LF99 | At5g65730 | 33.7 | **Ap, Cyt**, ER, G, Chl | Cell wall biogenesis, cell wall organization, response to water deprivation, xyloglucan metabolism | A | ↓ | YES | YES | ** |
| Probable alpha-mannosidase | | Q8LPJ3 | At5g13980 | 115.8 | **Ap, V**, **T**, Mit, PM, ER, G, Cyt | Mannose metabolism, protein deglycosylation |  | ↓ | YES | YES | ** |
| Eukaryotic aspartyl protease family protein | | Q8RX60 | At1g79720 | 52.4 | **Ap**, PM, V, N, ER, G | Proteolysis |  | ↓ | YES | YES | **** |
| Probable pectinesterase/pectinesterase inhibitor 41 (PE 41) | | Q8RXK7 | At4g02330 | 63.9 | **Ap**, Chl, ER, G, Mit | Cell wall modification, pectin catabolism, response to cold, response to fungus | B - A | ↓ | YES | YES | ** |
| Ferredoxin--NADP reductase, leaf isozyme 2 (FNR-2) | | Q8W493 | At1g20020 | 41.1 | **Ap, Mit, G, N, Chl**, Cyt | Defense response to bacteria, defense response to fungus, oxidation-reduction process, photosynthesis | B - A | ↓ | NO | YES | ** |
| GDSL esterase/lipase | | Q93YW8 | At4g18970 | 39.6 | **Ap**, Cyt, PX, N, PM, G, V, ER | Lipid catabolism |  | ↓ | YES | NO | ** |
| Beta-galactosidase 17 (BGAL17) | | Q93Z24 | At1g72990 | 78.6 | **Ap**, **V**, Cyt, Mit, Chl, G, ER | Carbohydrate metabolism |  | ↑ | YES | YES | ** |
| LL-diaminopimelate aminotransferase (DAP-AT) | | Q93ZN9 | At4g33680 | 50.4 | **Ap, Mit, PM, Chl** | Lysine biosynthetis, systemic acquired resistance, salicylic acid mediated signaling pathway |  | ↓ | NO | YES | *** |
| Fructose-bisphosphate aldolase 2 (AtFBA2) | | Q944G9 | At4g38970 | 43.0 | **Ap, G, Mit, N, PM, M, Chl, T** | Gluconeogenesis, glycolytic process, pentose-phosphate shunt |  | ↓ | NO | YES | **** |
| Peroxiredoxin-2E (PRXIIE) | | Q949U7 | At3g52960 | 24.7 | **Ap, G, Mit, Chl**, N, PX | Cell redox homeostasis, Q94JQ4response to oxidative stress, defense response to bacteria, hydrogen peroxide catabolism, oxidation-reduction process | A | ↓ | NO | YES | ** |
| Bifunctional purple acid phosphatase 26 (AtPAP26) | | Q949Y3 | At5g34850 | 55.0 | **Ap, Cyt, G, V**, ER | Dephosphorylation, hydrogen peroxide catabolism, oxidation-reduction process, phosphate ion homeostasis |  | ↑ | YES | YES | ** |
| unknown protein | | Q94A98 | At1g65900 | 45.2 | **Chl,** Ap, G, PM, V, ER | Unknown |  | ↓ | YES | YES | ** |
| Glycine dehydrogenase (decarboxylating) 1 (AtGLDP1) | | Q94B78 | At4g33010 | 112.9 | **Ap, Mit, Chl**, Cyt, V | Glycine catabolism, glycine decarboxylation, oxidation-reduction process |  | ↓ | NO | YES | ** |
| Reactive Intermediate Deaminase A (RIDA) | | Q94JQ4 | At3g20390 | 19.8 | **Ap, Mit, Chl**, **V**, Cyt | Branched-chain amino acid biosynthetic process, isoleucine biosynthesis, organonitrogen compound catabolism, response to toxic substance |  | ↓ | NO | NO | ** |
| Cyclase family protein | | Q94JT5 | At4g35220 | 30.0 | **AP, G, T, ER, Cyt,** PM, N, Mit, Chl | Tryptophan catabolism to kynurenine |  | ─ | YES | YES | ** |
| Myrosinase 2 (TGG2) | | Q9C5C2 | At5g25980 | 62.7 | **Ap, N, G, PX, PM, Chl, V**, Cyt, ER | Abscisic acid-activated signaling pathway, carbohydrate metabolism, defense response to insect, glucosinolate catabolism, regulation of stomatal movement, response to salt stress | B - A | ↓ | YES | YES | *** |
| 2-Cys peroxiredoxin BAS1-like (2-Cys Prx B) | | Q9C5R8 | At5g06290 | 29.8 | **Chl**, **Ap**, Cyt, Mit | Cell redox homeostasis, defense response to bacteria, hydrogen peroxide catabolism, oxidation-reduction process, response to cold, response to oxidative stress |  | ↓ | NO | YES | ** |
| Uncharacterized protein T8G24.2 | | Q9C6U3 | At3g08030 | 34.7 | **Ap, G, Mit, PM, T**, Chl, Cyt, ER | Unknown |  | ↓ | NO | YES | ** |
| GDSL esterase/lipase At1g29670 | | Q9C7N4 | At1g29670 | 39.8 | **Ap, G, Mit, PM, Chl, T**, ER | Lipid catabolism |  | ↓ | YES | NO | **** |
| GDSL esterase/lipase At1g29660 | | Q9C7N5 | At1g29660 | 40.1 | **Ap, Mit, N**, ER, Cyt, G | Lipid catabolism, systemic acquired resistance | B | ↓ | YES | NO | **** |
| Probable thionin-2.4 | | Q9C8D6 | At1g66100 | 14.1 | **Ap**, Chl, V, G, N, ER | Defense response, pathogenesis | B | ↓ | YES | YES | ** |
| Glycerate dehydrogenase HPR (AtHPR1) | | Q9C9W5 | At1g68010 | 42.2 | **Ap, Cyt, Mit, N, PX, PM, Chl** | Response to light, response to water deprivation, oxidation-reduction process, photorespiration |  | ↓ | NO | YES | ** |
| MD-2-related lipid-recognition protein 3 (ML3) | | Q9FF98 | At5g23820 | 17.9 | **Ap, T**, **V**, N, PM, G, ER, Mit | Defense response | B | ↓ | YES | NO | ** |
| Subtilisin-like protease SBT2.3 (AtSBT2.3) | | Q9FI12 | At5g44530 | 90.6 | **Ap**, G, Mit, PM, V, Chl, ER | Proteolysis |  | ↓ | YES | YES | ** |
| Pectin lyase-like superfamily protein | | Q9FJ27 | At5g41870 | 48.6 | **Ap**, G, Cyt, PM, Chl, ER | Carbohydrate metabolism, cell wall organization |  | ↑ | YES | YES | ** |
| Bifunctional inhibitor/lipid-transfer protein/seed storage 2S albumin superfamily protein | | Q9FJ65 | At5g55450 | 11.0 | **Ap, ER, Chl**, G, PM | Response to other organism, systemic acquired resistance | B | ↓ | YES | NO | **** |
| Carbohydrate-binding X8 domain superfamily protein | | Q9FKH4 | At5g35740 | 13.4 | **PM**, Ap, G, Chl, ER | Unknown |  | ↓ | YES | NO | ** |
| Ferredoxin--NADP reductase (LFNR1) | | Q9FKW6 | At5g66190 | 40.3 | **Ap, G, Mit, N, PM, Chl, T**, Cyt | Defense response to bacteria, oxidation-reduction process, photosynthetic electron transport chain |  | ↓ | NO | YES | ** |
| Fasciclin-like arabinogalactan protein 1 (FLA1) | | Q9FM65 | At5g55730 | 44.8 | **Ap, EV, G, PM**, **M**, Mit, N, ER | Root development, shoot system development |  | ↓ | YES | YES | **** |
| Alpha-galactosidase 1 (AtAGAL1) | | Q9FT97 | At5g08380 | 45.7 | **Ap**, ER, Mit, N, G, Cyt | Carbohydrate metabolism, cell wall organization |  | ↑ | YES | YES | ** |
| Leucine-rich repeat (LRR) family protein | | Q9FXA1 | At1g49750 | 54.4 | **Ap, G, Mit, N, PM, Chl**, ER, V | Unknown |  | ↓ | YES | YES | ** |
| Beta-glucosidase 40 (AtBGLU40) | | Q9FZE0 | At1g26560 | 58.1 | **Ap, Chl**, N, V, G, ER, Mit | Carbohydrate metabolism |  | ↓ | YES | YES | ** |
| Phosphoglycerate kinase 1 (PGK1) | | Q9LD57 | At3g12780 | 50.1 | **Ap, Cyt, G, Mit, N, PM, M, T, Chl** | Galactolipid biosynthesis, gluconeogenesis, glycolytic process, protein phosphorylation, positive regulation of oxidative phosphorylation, reductive pentose-phosphate cycle, response to cold, thylakoid membrane organization |  | ↓ | NO | YES | **** |
| Non-specific lipid-transfer protein 6 (LTP 6) | | Q9LDB4 | At3g08770 | 11.9 | **Ap**, **M**, ER, G, V | Lipid transport, response to water deprivation |  | ↓ | YES | NO | ** |
| Fructose-bisphosphate aldolase 8 (AtFBA8) | | Q9LF98 | At3g52930 | 38.5 | **Ap, Cyt, G, Mit, N, PM, Chl, T**, ER | Gluconeogenesis, glycolytic process, pentose-phosphate shunt, response to salt stress |  | ↓ | NO | NO | *** |
| Probable inactive purple acid phosphatase 2 (PAP2) | | Q9LMG7 | At1g13900 | 73.7 | **Ap, G, Chl, T, Mit**, **Cyt**, V, PM, N, ER | Protein targeting to mitochondrion, regulation of carbohydrate metabolism |  | ↑ | YES | YES | ** |
| Probable inactive purple acid phosphatase 1 (PAP1) | | Q9LMX4 | At1g13750 | 68.2 | **Ap**, N, V, ER, G, PM | Dephosphorylation |  | ↑ | YES | YES | ** |
| CO(2)-response secreted protease (AtSBT5.2) | | Q9LNU1 | At1g20160 | 81.4 | **Ap**, ER, G, V, PM | Negative regulation of defense response to bacteria, negative regulation of stomatal complex development, proteolysis, response to carbon dioxide |  | ↓ | YES | YES | **** |
| Glycine cleavage system H protein 3 (GDH3) | | Q9LQL0 | At1g32470 | 17.9 | **Mit, Chl** | Glycine decarboxylation |  | ↓ | NO | YES | ** |
| Glutamate--glyoxylate aminotransferase 1 (AtGGT2) | | Q9LR30 | At1g23310 | 53.3 | **Ap, EV, Mit, N, PX, PM, M Chl, V,** G, Cyt, ER, | L-alanine catabolism, glycine biosynthesis, photorespiration, response to hypoxia |  | ↓ | NO | YES | *** |
| (S)-2-hydroxy-acid oxidase GLO2 (GOX 2) | | Q9LRS0 | At3g14415 | 40.3 | **Ap, G, Mit, N, PX, PM, Chl, V, T**, Cyt | Defense response to bacteria, hydrogen peroxide biosynthesis, oxidation-reduction process, photorespiration | B | ↓ | NO | YES | ** |
| Leucine-rich repeat (LRR) family protein | | Q9LUN3 | At3g17640 | 42.9 | Ap, G, Mit, Chl, ER, V, N | Signal transduction |  | ↓ | YES | YES | ** |
| S-adenosylmethionine synthase 4 (METK4) | | Q9LUT2 | At3g17390 | 42.8 | **Ap, G, Mit, N, PM, M, Chl, V**, Cyt, ER | Lignin biosynthesis, methionine metabolism, one-carbon metabolism, response to cold |  | ↓ | NO | YES | ** |
| Encodes a protein with similarity to a lipid transfer protein that may contribute to systemic acquired resistance (SAR) | | Q9LV65 | At5g48490 | 10.7 | **Ap**, PM, G, ER | Systemic acquired resistance | B | ↓ | YES | NO | ** |
| Probable beta-D-xylosidase 6 (AtBXL6) | | Q9LXA8 | At5g10560 | 87.1 | **Ap, V**, **T**, Cyt, Mit, ER, G, N, Chl, PM | Carbohydrate metabolism |  | ↓ | YES | YES | **** |
| Soluble inorganic pyrophosphatase 6 (PPA6) | | Q9LXC9 | At5g09650 | 33.4 | **Ap, Mit, PM, M, Chl**, Cyt | Defense response to bacteria, phosphate-containing compound metabolism, response to salt stress | B - A | ↓ | NO | YES | ** |
| Probable pectinesterase/pectinesterase inhibitor 51 (PME51) | | Q9LXD9 | At5g09760 | 60.4 | **Ap**, **Chl**, Cyt, Mit, M, ER, G, V | Cell wall modification, pectin catabolism |  | ↓ | YES | YES | **** |
| Proline-tRNA ligase (DUF1680) | | Q9LXU4 | At5g12950 | 96.2 | **Ap**, **V**, ER, Mit, PM, Chl, G | unknown |  | ─ | YES | YES | ** |
| Alpha-glucosidase 1 | | Q9LYF8 | At5g11720 | 101.1 | **Ap, PM, V**, ER, G, Chl, Mit | Carbohydrate metabolism |  | ↓ | YES | YES | ** |
| Calvin cycle protein (CP12-2) | | Q9LZP9 | At3g62410 | 14.2 | **Chl**, N, Cyt, Mit | Response to anoxia, response to cold, response to heat, protein-containing complex assembly, negative regulation of reductive pentose-phosphate cycle, peptide cross-linking via L-cystine, response to light | A | ↓ | NO | YES | ** |
| Bifunctional inhibitor/lipid-transfer protein/seed storage 2S albumin superfamily protein | | Q9M329 | At3g53980 | 11.7 | **Ap**, PM, ER, G | Systemic acquired resistance | B | ─ | YES | NO | ** |
| GDSL esterase/lipase At3g05180 | | Q9MAA1 | At3g05180 | 42.3 | **Ap**, PM, G, ER, Chl | Lipid catabolism, systemic acquired resistance | B | ↓ | YES | YES | ** |
| Probable ribose-5-phosphate isomerase 3 (RPI3) | | Q9S726 | At3g04790 | 29.3 | **Ap, G, Mit, PM, Chl**, ER, Cyt | Defense response to bacteria, reductive pentose-phosphate cycle | B | ↓ | NO | NO | *** |
| Phosphoglycerate kinase 3 (PGK3) | | Q9SAJ4 | At1g79550 | 42.1 | **Ap, Cyt, G, N, PM, Chl, V, T,** Mit | Gluconeogenesis, glycolytic process, positive regulation of oxidative phosphorylation, response to heat, response to light, response to molecule of bacterial origin |  | ↓ | NO | YES | ** |
| Beta-galactosidase 3 (BGAL3) | | Q9SCV9 | At4g36360 | 95.1 | **Ap**, **V**, G, N, ER, Cyt, Mit, PM | Carbohydrate metabolism |  | ↓ | YES | YES | ** |
| Beta-galactosidase 1 (BGAL1) | | Q9SCW1 | At3g13750 | 93.6 | **Ap, N**, **V**, G, Mit, ER, PM, Chl | Carbohydrate metabolism |  | ↓ | YES | YES | ** |
| Peptidyl-prolyl cis-trans isomerase (FKBP13) | | Q9SCY2 | At5g45680 | 22.0 | **Chl**, Ap, N, Cyt | Protein peptidyl-prolyl isomerization |  | ↓ | NO | YES | ** |
| Thioredoxin M4 | | Q9SEU6 | At3g15360 | 21.2 | **Ap, Chl**, Mit, N | Cell redox homeostasis, glycerol ether metabolism, regulation of catalytic activity, oxidation-reduction process, regulation of carbohydrate metabolism, response to oxidative stress |  | ↓ | NO | YES | ** |
| Adenosine kinase 1 (ADK1) | | Q9SF85 | At3g09820 | 37.8 | **Ap, EV, Cyt, G, PM, M, Chl**, **N**, Mit, ER | AMP salvage, phosphorylation, viral process |  | ↓ | NO | YES | ** |
| Protein of unknown function | | Q9SFB1 | At3g08030 | 39.0 | **Ap, G, Mit, PM, T**, **Chl**, ER, Cyt | Unknown |  | ↓ | YES | YES | *** |
| Alpha-L-arabinofuranosidase 1 (ASD1) | | Q9SG80 | At3g10740 | 75.0 | **Ap, V, T**, G, PM, Cyt, ER | L-arabinose metabolism, xylan catabolism |  | ↑ | YES | YES | *** |
| AT1G76160 protein (SKU5 similar 5) | | Q9SGR6 | At1g76160 | 60.0 | **Ap, G, Mit, PM**, V, ER | Iron ion homeostasis, iron ion transport, oxidation-reduction process |  | ↓ | YES | YES | **** |
| Receptor-like kinase (TMK3) | | Q9SIT1 | At2g01820 | 101.9 | **Ap, PM**, M, N, ER, Chl, Cyt, G | Pollen development, protein phosphorylation, signaling |  | ↓ | YES | YES | *** |
| Fructose-bisphosphate aldolase 6 (FBA6) | | Q9SJQ9 | At2g36460 | 38.4 | **Ap, Cyt, Mit, PM, M, Chl, T, N** | Gluconeogenesis, glycolytic process, mitochondria-nucleus signaling pathway, pentose-phosphate shunt |  | ↑ | NO | NO | ** |
| Fructose-bisphosphate aldolase 1 (FBA1) | | Q9SJU4 | At2g21330 | 42.9 | **Ap, G, Chl, T**, Mit, N | Gluconeogenesis, glycolytic process, pentose-phosphate shunt |  | ↓ | NO | YES | *** |
| Probable steroid-binding protein 3 (MP3) | | Q9SK39 | At2g24940 | 11.0 | **Ap, Cyt, PM, Chl**, **M**, **N**, Mit | Unknown |  | ↓ | NO | YES | ** |
| Bifunctional inhibitor/lipid-transfer protein/seed storage 2S albumin superfamily protein | | Q9SKI0 | At2g10940 | 29.6 | **Ap, G, Mit, N, PM, M, Chl, T**, PX, ER, V | Unknown |  | ↓ | YES | NO | *** |
| Triosephosphate isomerase (TIM) | | Q9SKP6 | At2g21170 | 33.3 | **Ap, Cyt, Mit, PM, Chl** | Chloroplast organization, gluconeogenesis, glyceraldehyde-3-phosphate biosynthesis, glycerol catabolism, glycolytic process, primary root development, reductive pentose-phosphate cycle, triglyceride mobilization |  | ↓ | NO | YES | ** |
| Cytosolic isocitrate dehydrogenase [NADP] (CICDH) | | Q9SRZ6 | At1g65930 | 45.7 | **Ap, EV, Cyt, G, PM, Chl, T**, V Mit, ER, N | NADP metabolism, defense response to bacteria, isocitrate metabolism, tricarboxylic acid cycle, response to salt stress, oxidation-reduction process | B - A | ↓ | NO | YES | ** |
| Monocopper oxidase-like protein (SKU5) | | Q9SU40 | At4g12420 | 65.6 | **Ap, EV, G, PM, V**, ER, Cyt | Cell tip growth, iron ion homeostasis, iron ion transport, oxidation-reduction process |  | ↓ | YES | YES | ** |
| Trehalase (TRE1) | | Q9SU50 | At4g24040 | 71.3 | **PM,** M, Ap, Mit, Chl, G, ER | Trehalose catabolism |  | ↑ | NO | YES | ** |
| Polyketide cyclase/dehydrase and lipid transport superfamily protein | | Q9SUR0 | At4g23670 | 17.5 | **Ap, EV, PM, Chl, V**, Cyt, Mit, N | Defense response to bacteria, response to salt stress | B - A | ↓ | NO | YES | ** |
| Berberine bridge enzyme-like 21 (AtBBE-like 21) | | Q9SVG3 | At4g20840 | 60.1 | **Ap, PM**, **Chl**, ER, Mit, Cyt, G, PX | Oxidation-reduction process | A | ↓ | YES | YES | ** |
| GDSL esterase/lipase. | | Q9SVU5 | At4g28780 | 39.9 | **Ap**, G, Cyt, Chl, V, Mit, ER, N, PM | Lipid catabolism |  | ↓ | YES | YES | ** |
| Annexin D1 (AnnAt1) | | Q9SYT0 | At1g35720 | 36.2 | **Ap, EV, Cyt, Mit, N, PX, PM, Chl, V, T, M,** G, ER | Calcium ion transmembrane transport, phloem sucrose unloading, primary root development, response to cold, response to heat, response to osmotic stress, response to oxidative stress, response to salt stress, response to water deprivation | A | ↓ | NO | YES | ** |
| Peroxidase 51 (PRX51) | | Q9SZE7 | At4g37530 | 36.0 | **Ap**, ER, V, N, G, Cyt | Hydrogen peroxide catabolism, oxidation-reduction process, response to oxidative stress |  | ↑ | YES | YES | *** |
| Thioredoxin F1 (AtTrxf1) | | Q9XFH8 | At3g02730 | 19.3 | **Mit, Chl**, Cyt | Cell redox homeostasis, regulation of catalytic activity, oxidation-reduction process, regulation of carbohydrate metabolism, response to light intensity |  | ↓ | NO | YES | ** |
| Involved in response to salt stress | | Q9XI93 | At1g13930 | 16.2 | **EV, PM**, **Chl**, Mit, N, Cyt | Regulation of abscisic acid biosynthesis, response to heat, response to salt stress |  | ↓ | NO | YES | *** |
| Ferredoxin-dependent glutamate synthase 1 (Fd-GOGAT 1) | | Q9ZNZ7 | At5g04140 | 176.6 | **Ap, Mit, G, Chl**, **M**, Cyt | L-glutamate biosynthesis, Ammonia assimilation cycle, oxidation-reduction process, photorespiration, positive regulation of glycine hydroxymethyltransferase activity, response to light |  | ↓ | NO | YES | *** |
| Malate dehydrogenase 1 (MDH1) | | Q9ZP06 | At1g53240 | 35.8 | **Ap, G, Mit, PM, T, Chl** | Carbohydrate metabolism, defense response to bacteria, malate metabolism, response to cold, response to salt stress, tricarboxylic acid cycle | B - A | ↓ | NO | YES | ** |
| Putative uncharacterized protein At1g09310 | | Q9ZPZ4 | At1g09310 | 19.9 | **Ap, EV, PM, Chl**, **N**, Mit, Cyt | Unknown |  | ↓ | NO | NO | ** |
| Cysteine proteinase | | Q9ZQH7 | At2g27420 | 38.7 | **Ap**, ER, PM, G, Cyt, Chl, V | Proteolysis |  | ↓ | YES | YES | ** |
| NADPH-dependent alkenal/one oxidoreductase (AOR) | | Q9ZUC1 | At1g23740 | 41.0 | **Ap, Chl, Mit**, Cyt | Oxidation-reduction process, response to cold | A | ↓ | NO | YES | *** |
| Non-specific lipid-transfer protein (LTP6) | | F4IXC6 | At3g08770 | 12.3 | **Ap**, **M**, G, ER, V | Lipid transport, response to water deprivation | A | ↓ | YES | NO | ** |
| Probable mediator of RNA polymerase II transcription subunit 37e (AtHsp70-1) | | P22953 | At5g02500 | 71.3 | **Ap, EV, Cyt, G, Mit, N, PX, PM, M, Chl, V, T,** ER | Defense response to bacteria, defense response to fungus, negative regulation of seed germination, protein folding, protein refolding, response to cold, response to heat, response to virus, stomatal closure | B - A | ↓ | NO | YES | ** |
| Superoxide dismutase [Cu-Zn] 1 (CSD1) | | P24704 | At1g08830 | 15.1 | **Ap, Mit, Cyt, PM**, N, ER, Chl, G | Response to UV-B, response to light, defense response to bacteria, gene silencing by miRNA, oxidation-reduction process, removal of superoxide radicals, response to oxidative stress, response to ozone, response to salt stress | A | ↓ | NO | YES | ** |
| Nucleoside diphosphate kinase 1 (NDK1) | | P39207 | At4g09320 | 16.5 | **Ap, EV, Cyt, G, Mit, PX, PM, Chl, V, N**, ER | Biosynthesis of CTP, GTP and UTP, response to hydrogen peroxide, nucleoside diphosphate phosphorylation, |  | ↓ | NO | NO | ** |
| Presequence protease 1, chloroplastic/mitocondrial (PREP1) | | Q9LJL3 | At3g19170 | 120.9 | **Ap, Mit, Chl, G,** Cyt | Protein processing, proteolysis |  | ↓ | NO | YES | ** |
| Heat shock 70 kDa protein 6 (HSP70-6) | | Q9STW6 | At4g24280 | 76.5 | **Ap, G, Mit, N, PM, Chl, T** | Protein folding, , protein targeting to chloroplast, responses to cold, heat, viral process | A | ↓ | NO | YES | ** |
|  | **Supplementary Table S3. AF proteins whose levels decrease significantly more than twofold during leaf senescence.**  Four biological replicas for each leaf stage were analyzed. A confidence level (CL) was assigned to each protein based on the number of biological replicates in which it appears: **, present in two biological replicas; ***, present in three biological replicas and ****, present in the four biological replicas. Rows highlighted in gray: proteins detected only in AF S2 (absent in AF S3).  A confidence level (CL) was assigned to each protein based on the number of biological replicates in which it appears: **, present in two biological replicas; ***, present in three biological replicas and ****, present in the four biological replicas. Rows highlighted in gray: proteins detected only in AF S3. Gene expression patterns associated with senescence (column "gene expression during senescence") were examined using the eFP Browser software (Winter et al., 2007). Subcellular locations and protein functions were determined using the SUBA4 and Gene Ontology databases. In bold, manually-assigned locations and in normal font, locations that were inferred from electronic annotation (IEA) or predicted. Biotic (B) or abiotic (A) stress related function were assigned according to Gene Ontology, or otherwise experimental data from literature. Presence of SP was determined by the SignalP 4.1 software , and presence of potential N-glycosylation sites was determined with the ScanProsite tool .  Abbreviations: ID=identification, SL= subcellular localization, SP= signal peptide, N-g.s.= potential N-glycosylation sites, CL= confidence level, M=membrane, PM=plasma membrane, G=Golgi, N=Nucleo, Cyt=cytoplasm, Chl=chloroplast, Mit=mitochondrion, Ap=apoplast, ER= endoplasmic reticulum, PX=peroxisome, V=vacuole, T=tonoplast, EV= extracellular vesicles | | | | | | | | | | |

Supplementary Table S4

| **Protein** | **Protein ID** | **Gen ID** | **MW (kDa)** | **SL** | **gene expression during senescence** | **SP** | **N-g.s.** | **CL** |  |
| --- | --- | --- | --- | --- | --- | --- | --- | --- | --- |
| Leucine-rich repeat protein kinase family protein | F4IB63 | At1g51805 | 98.1 | **PM, V, T,** M, Chl, N, Cyt, G, ER | ↑ | YES | YES | ** |  |
| Pyridoxamine 5'-phosphate oxidase family protein | F4IFA9 | At2g04690 | 22.8 | **Ap, V,** PM, N, G, ER, Mit, Chl | ↑ | YES | NO | ** |  |
| Fasciclin-like arabinogalactan protein 8 (FLA8) | O22126 | At2g45470 | 43.1 | **Ap, Chl, PM,** G, ER, Mit, V | ↓ | YES | YES | **** |  |
| Peroxidase 3 (RCI3) | O23044 | At1g05260 | 34.9 | **Ap, ER,** Chl, G, N, Cyt | ─ | YES | YES | *** |  |
| Probable pectinesterase/pectinesterase inhibitor 12 (PME12) | O48711 | At2g26440 | 60.4 | **Ap,** Chl, G, ER, Mit, V | ↑ | YES | YES | *** |  |
| Protein YLS3 | O64864 | At2g44290 | 21.6 | **PM**, **Ap, M,** Chl, ER, Cyt, V, G, N | no inf. | YES | YES | *** |  |
| Uclacyanin-2 (UCC2) | O80517 | At2g44790 | 20.4 | **PM, Ap, G,** N, ER | ─ | YES | YES | *** |  |
| Pathogenesis-related protein 1 (PR1) | P33154 | At2g14610 | 17.7 | **Ap,** V, G, ER, Chl | ↑ | YES | YES | *** |  |
| Cysteine proteinase RD21A | P43297 | At1g47128 | 50.7 | **Ap, Chl, V, G, T,** ER | ↑ | YES | YES | ** |  |
| Cysteine proteinase inhibitor 5 (CYS5) | Q41916 | At5g47550 | 13.4 | **Ap,** ER, PM, G, Chl, V, Mit | ↓ | YES | YES | *** |  |
| Peroxidase 71 (PRX71) | Q43387 | At5g64120 | 34.9 | **Ap, M, G, T, PM,** Chl, ER, Cyt | ↓ | YES | YES | **** |  |
| Non-specific lipid-transfer protein-like protein (XYP1) | Q8VYI9 | At5g64080 | 18.0 | **M, PM,** ER, Chl, G, Ap | ↓ | YES | YES | ** |  |
| LysM domain-containing GPI-anchored protein 1 (LYP2) | Q93ZH0 | At1g21880 | 43.5 | **Ap, M, PM,** G, ER, Cyt | ↓ | YES | YES | **** |  |
| Auxin-induced in root cultures protein 12 (AIR12) | Q94BT2 | At3g07390 | 25.6 | **PM, Ap, M,** Chl, ER, Mit, G, V, N | ↓ | YES | YES | **** |  |
| Glucan endo-1,3-beta-glucosidase 4 | Q94CD8 | At3g13560 | 54.4 | **PM, M, Ap,** G, ER, Mit, V, Chl | ↓ | YES | YES | ** |  |
| Fasciclin-like arabinogalactan protein 13 (FLA13) | Q9FFH6 | At5g44130 | 26.2 | **Ap, G, M, PM,** ER, Mit | ↓ | YES | YES | **** |  |
| Probable LRR receptor-like serine/threonine-protein kinase | Q9FN93 | At5g59680 | 98.4 | **M, PM, Ap,** G, Mit, N, ER | ─ | YES | YES | **** |  |
| Glutathione S-transferase DHAR1, mitocondrial | Q9FWR4 | At1g19570 | 23.6 | **Chl, Mit, V, Ap, PX, Cyt, M, PM,** G, N, ER | ─ | NO | YES | **** |  |
| Heparanase-like protein 3 | Q9FZP1 | At5g34940 | 59.7 | **Ap,** Mit, Cyt, N, ER, G | ↓ | YES | YES | **** |  |
| Aspartyl protease AED1 | Q9LEW3 | At5g10760 | 49.5 | **Ap,** PM, Chl, ER, V, G, N | ↑ | YES | YES | **** |  |
| Protein ASPARTIC PROTEASE IN GUARD CELL 1 (ASPG1) | Q9LS40 | At3g18490 | 53.2 | **Ap, ER, PM, G,** Chl, Mit, V, N | ↑ | YES | YES | **** |  |
| Glucan endo-1,3-beta-glucosidase 7 | Q9M069 | At4g34480 | 53.1 | **Ap, PM,** G, V, ER, N, Mit, Chl | ↑ | YES | NO | *** |  |
| Glucan endo-1,3-beta-glucosidase 5 | Q9M088 | At4g31140 | 52.7 | **M, PM, V, Ap, T,**  G, ER | ─ | YES | YES | *** |  |
| Fasciclin-like arabinogalactan protein 7 (FLA7) | Q9SJ81 | At2g04780 | 26.8 | **M, PM, Ap,** N, ER, G | ↓ | YES | YES | *** |  |
| At2g15220 | Q9SKL6 | At2g15220 | 25.2 | **Ap,** G, ER, Mit, Cyt | ↑ | YES | NO | *** |  |
| Fasciclin-like arabinogalactan protein 2 (FLA2) | Q9SU13 | At4g12730 | 43.4 | **Ap, M, PM, T, V,** Chl, ER, G, Mit | ↓ | YES | YES | *** |  |
| Cystine lyase CORI3 | Q9SUR6 | At4g23600 | 47.0 | **Cyt, V, Ap, Mit, Chl, PM,** ER, G | ↓ | NO | NO | *** |  |
| DPP6 amino-terminal domain protein | Q9XI11 | At1g21670 | 77.3 | **Ap,** Mit, Chl, ER, G, V, Cyt | ↑ | YES | YES | ** |  |
| Putative protease inhibitor | Q9ZV18 | At2g38870 | 7.6 | **Ap,** N, Mit, Cyt, Chl, ER, G | ↓ | NO | NO | *** |  |
| **Supplementary Table S4. AF proteins whose levels do not change significantly during leaf senescence.**  Four biological replicas for each leaf stage were analyzed. A confidence level (CL) was assigned to each protein based on the number of biological replicates in which it appears: **, present in two biological replicas; ***, present in three biological replicas and ****, present in the four biological replicas.  Gene expression patterns associated with senescence (column "gene expression during senescence") were examined using the eFP Browser software (Winter et al., 2007).  Subcellular locations and potential functions were determined using the SUBA4 databases (Hooper et al., 2016), UniProt KB (The UniProt Consortium, 2017) and Gene Ontology (Ashburner et al., 2000; Consortium, 2016). In bold, manually-assigned locations and in normal font, locations that were inferred from electronic annotation (IEA) or predicted. Presence of SP was determined by the SignalP 4.1 software , and presence of potential N-glycosylation sites was determined with the ScanProsite tool .  Abbreviations: ID=identification, SL= subcellular localization, SP= signal peptide, N-g.s.= probable N-glycosylation sites, CL= confidence level, M=membrane, PM=plasma membrane, G=Golgi, N=Nucleo, Cyt=cytoplasm, Chl=chloroplast, Mit=mitochondrion, Ap=apoplast, ER= endoplasmic reticulum, PX=peroxisome, V=vacuole, T=tonoplast, EV= extracellular vesicles . | | | | | | | | | |
